# Internet Treatment for Depression: A Randomized Controlled Trial Comparing Clinician vs. Technician Assistance

**DOI:** 10.1371/journal.pone.0010939

**Published:** 2010-06-08

**Authors:** Nickolai Titov, Gavin Andrews, Matthew Davies, Karen McIntyre, Emma Robinson, Karen Solley

**Affiliations:** 1 Clinical Research Unit for Anxiety and Depression, School of Psychiatry, University of New South Wales at St Vincent's Hospital, Sydney, New South Wales, Australia; 2 Clinical Research Unit for Anxiety and Depression, St Vincent's Hospital, Sydney, New South Wales, Australia; University of Granada, Spain

## Abstract

**Background:**

Internet-based cognitive behavioural therapy (iCBT) for depression is effective when guided by a clinician, less so if unguided. Question: Would guidance from a technician be as effective as guidance from a clinician?

**Method:**

Randomized controlled non-inferiority trial comparing three groups: Clinician-assisted vs. technician-assisted vs. delayed treatment. Community-based volunteers applied to the VirtualClinic (www.virtualclinic.org.au) research program, and 141 participants with major depressive disorder were randomized. Participants in the clinician- and technician-assisted groups received access to an iCBT program for depression comprising 6 online lessons, weekly homework assignments, and weekly supportive contact over a treatment period of 8 weeks. Participants in the clinician-assisted group also received access to a moderated online discussion forum. The main outcome measures were the Beck Depression Inventory (BDI-II) and the Patient Health Questionnaire-9 Item (PHQ-9). Completion rates were high, and at post-treatment, both treatment groups reduced scores on the BDI-II (p<0.001) and PHQ-9 (p<0.001) compared to the delayed treatment group but did not differ from each other. Within group effect sizes on the BDI-II were 1.27 and 1.20 for the clinician- and technician-assisted groups respectively, and on the PHQ-9, were 1.54 and 1.60 respectively. At 4-month follow-up participants in the technician group had made further improvements and had significantly lower scores on the PHQ-9 than those in the clinician group. A total of approximately 60 minutes of clinician or technician time was required per participant during the 8-week treatment program.

**Conclusions:**

Both clinician- and technician-assisted treatment resulted in large effect sizes and clinically significant improvements comparable to those associated with face-to-face treatment, while a delayed treatment control group did not improve. These results provide support for large scale trials to determine the clinical effectiveness and acceptability of technician-assisted iCBT programs for depression. This form of treatment has potential to increase the capacity of existing mental health services.

**Trial Registration:**

Australian New Zealand Clinical Trials Registry ACTRN12609000559213

## Introduction

Depression and anxiety are both common and disabling [1]–[3]. Although effective treatments such as medication and cognitive behavioural therapy (CBT) are available, less than half report seeking any treatment and few receive specialized treatment from trained mental health professionals [4]. Barriers to effective treatment include limited availability of skilled clinicians, direct and indirect costs of treatment, and difficulty attending treatment during office hours.

One strategy for reducing barriers to effective treatment is the development of computerized cognitive behavioural therapy (CCBT) programs, which present structured sessions of CBT via a computer interface [5], [6]. Such programs may be accessed by patients at primary or specialist care facilities, via the Internet or DVD, and may be completed with the assistance of a clinician or entirely self-guided. CCBT reduces the demands on clinician time but the results of entirely self-guided treatment has been significantly inferior [7]–[9].

A growing body of evidence indicates that clinician-assisted Internet-based CBT (iCBT) can result in significant improvements in patients with depressive [9]–[13] and anxiety disorders [14]–[27], with results comparable to those obtained from good face-to-face treatment. Recent studies of iCBT programs for generalized anxiety disorder and social phobia indicate that clinician time can be reduced considerably by delegating the majority of patient contact to a non-clinician (technician), without compromising clinical outcomes or acceptability [16], [28], [29]. iCBT programs that can be safely and effectively administered by technicians who are supervised by clinicians, have considerable potential for enhancing existing mental health services. An important question is whether similar effects could be obtained from iCBT for depression when administered by a technician.

The present CONSORT-Revised compliant randomized controlled trial (RCT) [30] examined the clinical efficacy and acceptability of clinician- and technician-assisted iCBT using the *Sadness* program [12], [13] for people with a DSM-IV [31] diagnosis of depression. We hypothesised that participants in a technician-assisted (TA) group would show similar clinical improvements on measures of depression and disability as a clinician-assisted (CA) group; that improvements would be sustained at follow-up; and that both treatment groups would have better outcomes than a delayed treatment (control) group.

## Methods

The protocol for this trial and supporting CONSORT checklist are available as supporting information; see Checklist S1 and Protocol S1.

### Ethics

This study was approved by the St Vincent's Hospital Human Research Ethics Committee (HREC) and by the University of New South Wales HREC. Written informed consent was obtained from all participants.

### Participants

Participants were recruited from June to July 2009 via a website (www.virtualclinic.org.au) providing information about common mental disorders including depression, and a link to apply online to join a research treatment program. Participants first applied online, completing questionnaires about severity of symptoms, demographic details, and chronicity of symptoms (see Table 1).

**Table 1:** Demographic description of participants.

		*Technician-Assisted*		*Clinician-Assisted*		*Control Group*		*Total*	
		(n = 41)		(n = 45–46) *		(n = 40)		(n = 126–127)	
Variable	Sub-variable	*n*	%	*n*	%	*n*	%	*n*	%
Gender	Male	15	36.5	6	13.0	12	30.0	33	26.0
	Female	26	63.4	40	86.9	28	70.0	94	74.0
Age	Mean Age (SD)	44 (12.28)		40 (12.33)		46 (13.55)		43 (12.86)	
	Range	23–66		20–64		19–73		19–73	
Marital Status	Single/Never Married	10	24.4	17	36.9	11	27.5	38	29.9
	Married/De Facto	18	43.9	20	43.5	22	55	60	47.3
	Separated/Divorced	13	31.7	9	19.6	7	17.5	29	22.8
Education	High school	4	9.75	9	19.6	7	17.5	20	15.7
	Tertiary	27	65.85	26	56.5	24	60.0	77	60.6
	Other Certificate	9	21.95	10	21.7	9	22.5	28	22.04
	None	1	2.43	1	2.2	0	0	2	1.57
Employment Status	Part time/student	10	24.4	20	41.3	17	42.5	47	37.0
	Full time	18	43.9	13	30.4	15	37.5	46	36.2
	Unemployed, retired or disabled	13	31.7	13	28.3	8	20.0	34	26.8
Previously Discussed Symptoms with Health Professional		33	80.5	40	87.0	30	75.0	103	81.1
Taking Medication		26	63.4	26	46.5	22	55.0	74	58.3
Hours/Week of Internet use.	0–10	19	46.3	23	50.0	19	47.5	61	48.0
	11+	22	53.7	23	50.0	21	52.5	66	52.0
Confidence using computers and Internet	Very Confident	24	58.5	25	55.6	22	55.0	71	55.9
	Confident	8	19.5	16	35.6	9	22.5	33	26.9
	Average	7	17.1	2	4.4	7	17.5	16	12.6
	Mildly Confident	2	4.9	2	4.4	2	5.0	6	4.7
	Not Confident	0	0.0	0	0.0	0	0.0	0	0.0
History of symptoms of chronicity/severity of depression: Age of onset	<12	6	14.6	5	10.9				
	13–21	18	43.9	23	50.0				
	>22	12	29.3	13	28.3				
	Did not respond	5	12.2	5	10.9				
Number of episodes of depression?	1	3	7.3	2	4.3				
	2–4	5	12.2	7	15.2				
	5–8	6	14.6	10	21.7				
	>8	22	53.7	22	47.8				
	Did not respond	5	12.2	5	10.9				
Symptom free for 2 months or more in past 2 years?	Yes	6	14.6	15	32.6				
	No	30	73.2	25	54.3				
	Did not respond	5	12.2	6	17.4				

Exclusions were (i) not a resident of Australia; (ii) less than 18 years of age; (iii) no regular access to a computer, the Internet, and use of a printer; (iv) currently participating in CBT; (v) using illicit drugs or consuming more than three standard drinks/day; (vi) experience of a psychotic mental illness (schizophrenia or bipolar disorder) or current severe symptoms of depression (defined as a total score >23 or responding >2 to Question 9 (suicidal ideation) on the Patient Health Questionnaire - 9 Item (PHQ-9) [32]; (vii) a total PHQ-9 score below 10; and (viii) if taking medication, had been taking the same dose for less than 1 month or intending to change that dose during the course of the program. Excluded applicants immediately received an on-screen message and email thanking them for their application, and encouraging them to discuss their symptoms with their physician.

Participants who passed the screening phase were telephoned for a diagnostic interview using the Mini International Neuropsychiatric Interview Version 5.0.0 (MINI) [33] to determine whether they met DSM-IV criteria for major depressive episode. Participants who satisfied all criteria were informed of the study design and invited to return a completed consent form by email. The study was approved by the Human Research Ethics Committees of St Vincent's Hospital, Sydney, and the University of New South Wales.

### Interventions

Treatment group participants received access to the Sadness program, an iCBT program with demonstrated efficacy at reducing symptoms of depression [12]–[13]. The Sadness program consists of six online lessons, printable summary and homework assignments, automatic emails, and additional resource documents. The six online lessons represent best practice principles used in CBT programs for depression including behavioural activation, cognitive restructuring, problem solving, and assertiveness skills. Part of the content of each lesson is presented in the form of an illustrated story about a woman with depression who, with the help of a clinical psychologist, learns to gain mastery over her symptoms. Automatic emails are sent to congratulate participants for completing each lesson, to remind them to complete materials, and to notify them of new resources. As people progress through each lesson they have access to additional written documents providing supplementary information about techniques such as managing sleep problems, panic, and other common comorbid symptoms. They are also provided with access to vignettes written by previous participants about their own experiences of managing depression during the Sadness program. Participants are expected to complete the homework tasks prior to completing the next lesson, and to complete all lessons within 8 weeks.

All participants began the 8-week treatment program at the same time. Participants were advised to complete one lesson every 7–10 days and to complete the six lessons within 8 weeks of starting. All participants received automatic emails informing them when a lesson was to be completed, and reminder emails if they had not completed a lesson within 7 days of notification.

Three staff conducted the study, with supervision from NT. The technician (KM) was employed in an administrative role as a Clinic Manager, Anxiety Disorders Clinic, Mental Health Service, St Vincent's Hospital, Sydney. She reported no prior experience with research programs, no qualifications in health care or counseling, and had no clinical duties in her usual role. The clinician (MD) was a qualified and registered psychiatrist, employed at the same unit as KM. The third staff member (KS) was a research assistant, who provided administrative support to the technician and clinician.

#### Technician-Assisted Treatment

During treatment the technician provided TA group participants with weekly email or telephone contact. The technician was given a guideline script which identified the topics covered in each lesson of the program and activities participants should be encouraged to practice for each lesson. The technician was instructed to contact each TA group participant weekly to provide encouragement and support, and where possible to respond to participants' general questions by referring them to the materials in the Sadness program. The technician was not permitted to provide clinical advice. The technician received supervision from the clinician and was instructed to inform the clinician of any perceived deterioration in their participants' mental health status, or of any concerns about participants' wellbeing. While conducting this research the technician maintained her full-time role as a Clinic Manager.

#### Clinician-Assisted Treatment

CA group participants had weekly email or telephone contact with the clinician and access to an online discussion forum where they could post questions to the clinician about the program content. Information posted on the discussion forum could be read by other participants in the CA group. The clinician was provided with the same guideline script as the technician but was also instructed to answer participants' questions via forum, email, or telephone. The clinician was instructed to actively engage with each participant in treatment including goal setting, problem solving, and discussion of strategies for overcoming hurdles to progress. Because of the clinical nature of messages on the forum, the TA group did not have access to a forum. The clinician and technician were instructed to spend no more than 10 minutes in contact with each participant per week. The total time required and nature of all contacts with participants during treatment was recorded.

#### Control Group

Control group participants received the clinician-assisted program described above, but began treatment after the intervention groups completed the Sadness program.

### Objectives

This study was a 3 group randomized controlled non-inferiority trial to determine whether technician-assisted Internet treatment was equivalent to clinician-assisted Internet treatment but superior to delayed treatment (waitlist control).

### Primary and Secondary Outcomes

#### Outcomes

One week prior to beginning the trial participants completed the following questionnaires online: The PHQ-9, the Beck Depression Inventory (second edition) (BDI-II) [34], the Kessler 10 (K-10) [35], the Sheehan Disability Scales (SDS) [36], and the Credibility/Expectancy Questionnaire (CEQ) [37], [38]. The PHQ-9 is a 9-item measure with scores ranging from 0–27. The BDI-II is a 21-item measure with scores ranging from 0–64. A score of 10 on the PHQ-9 has been identified as providing an important threshold for identifying DSM-IV congruent depression [32], [39]. The K-10 is a 10-item measure of psychological distress with scores ranging from 10–50. The SDS is a 3 item measure of disability with scores ranging from 0–30 and the CEQ is a widely used measure of the expectancies or perception of treatment credibility.

The PHQ-9, BDI-II, K-10 and SDS were re-administered one week post-treatment and at four month post-treatment (follow-up), while the PHQ-9 was also administered mid-treatment (at week 5). All of these measures are considered reliable, valid, and appropriate for clinical and research purposes, with recent research indicating that online administration of questionnaires results in acceptable reliability of responses [40], [41]. Changes in the PHQ-9 and BDI-II were considered the primary outcome measures, while changes in the K-10, SDS and a treatment satisfaction questionnaire (based on the CEQ) were the secondary outcome measures. Follow-up results were not available for the control group, who had started treatment by that time.

### Sample Size and Randomization

Power calculations were based on a non-inferiority trial design comparing parallel-groups. Alpha was set at 0.025, power at 90%, and the mean minimal reliable change index for the PHQ-9 (based on earlier findings) and standard deviations for each group were expected to be equivalent (5 and 4, respectively). Using Table V from Julious [42], the minimum sample size for each group was identified as 39, but more were recruited to hedge against attrition.

**Figure 1:**
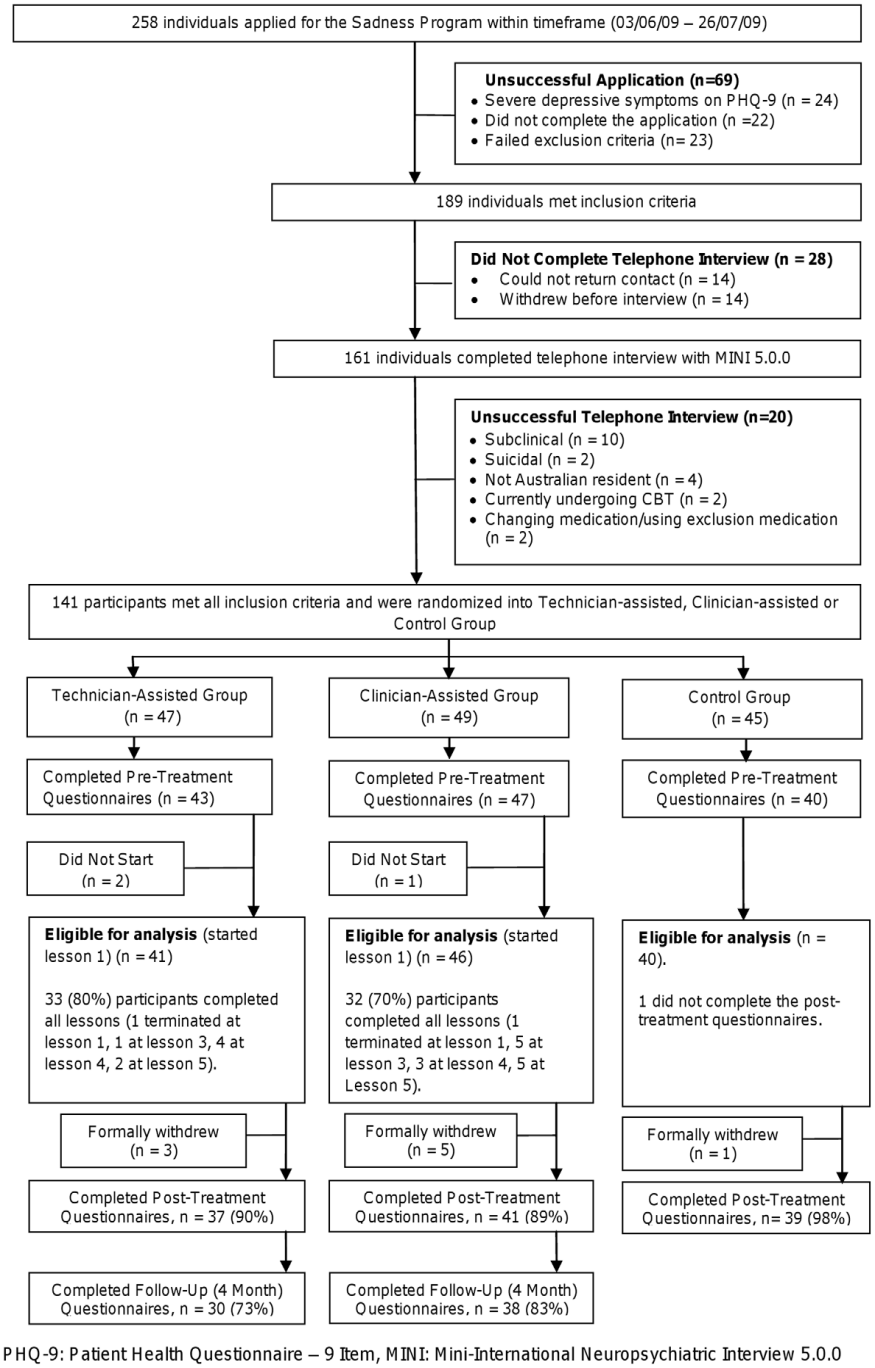
CONSORT-R participant flow chart.

The 141 people accepted into the program were randomised by NT via a true randomisation process (www.random.org) to either the CA (n = 49), TA (n = 47), or control groups (n = 45) (see Figure 1). Allocation preceded the diagnostic telephone call, and self report measures precluded blinding.

### Statistical Analysis

Group differences in demographic data, pre-treatment measures, and pre-treatment expectations were analyzed with one-way analysis of variance (ANOVAs) and chi-square tests, followed by *t*-tests with Bonferonni corrected *p* values. Changes in participants' questionnaire scores from pre to post-treatment and from pre-treatment to follow-up were analyzed using repeated measures analyses of covariance (ANCOVAs). This approach is recommended as a robust and reliable statistical strategy for analyzing the results of RCTs [43], [44]. Changes in questionnaire scores between post-treatment and follow-up were analyzed using paired samples *t*-tests. Effect sizes (Cohen's *d*) were calculated both within- and between-groups, based on the pooled standard deviation. We also calculated 95% confidence intervals for the between-group effect sizes between the two treatment groups for each primary outcome measure. All post-treatment and follow-up analyses adopt an intention-to-treat (ITT) design where missing data is replaced by the last observation carried forward (LOCF).

Two scores (credibility and expectancy) were derived from the CEQ by subjecting the scores to a linear transformation as described in [37]. Change scores for the two primary outcome measures, from pre to post and from pre to follow-up, were calculated for each intervention group participant. Pearson product moment correlations were calculated between these change scores and the total number of contacts with participants during the program and with the total time spent per participant, to explore variables that may affect outcomes.

Three measures of clinical significance were employed. First, pre-treatment and post-treatment PHQ-9 scores were compared with optimum cut-offs for a probable diagnosis of depression using evidence from the literature [32], [39], to provide an index of *remission*. This was defined as the proportion of participants who initially scored above the optimum cut-off (PHQ-9 total score of 10 or more) and subsequently scored below this cut-off. Secondly, an estimate of *recovery* was made by identifying the proportion of participants in each group who demonstrated a significant reduction in their symptoms (defined here, as a reduction of 50% of pre-treatment PHQ-9 scores) as described in recent dissemination studies [45]. Thirdly, the percentage of participants in each group who met criteria for both reliable and clinically significant change on the BDI-II was calculated. This was defined as the proportion of participants who met the criteria of statistically reliable change as described in Jacobson and Truax [46] and who also demonstrated a reduction in BDI-II scores of at least 10 points, a magnitude of change that has been used in similar research [47]. A reliable change index for the BDI-II was calculated separately for each of the three groups using their pre-treatment standard deviation, and a test-retest reliability coefficient of 0.93, as reported in the BDI-II manual [34].

## Results

### Participant Flow

Two hundred and fifty eight individuals expressed interest in the study (Figure 1), 141 met all inclusion criteria, and were randomized to one of the three groups. Forty one TA and 46 CA group participants completed the pre-treatment questionnaires and began lesson 1 and are eligible for analysis, as are 40 control group participants, who completed the pre-treatment questionnaires.

### Baseline Characteristics

Characteristics of the groups are presented in Table 1. There were no significant between-group differences in age, marital status, education, employment, previous discussions of symptoms with a health professional, use of medication, weekly use of the internet, confidence in using computers, treatment expectancies, or pre-treatment scores on the outcome measures. Treatment groups were also equally chronic, with most treatment group participants reporting at least 5 previous episodes of depression and fewer than 2 symptom free months in the past 2 years. However, there were significantly fewer males in the CA group (χ^2^ = 6.98, p<0.05).

### Completion Rates

Thirty three (33/41, 80%) TA and 32/46 CA (70%) group participants completed all 6 lessons within the required time. Post-treatment data was collected from 37/41 (90%) TA, 41/46 (89%) CA, and 39/40 (98%) control group participants. Follow-up data (4 months post-treatment) were collected from 30/41 (73%) TA and 38/46 (83%) CA group participants. In accordance with the intention-to-treat and LOCF models, missing post-treatment or follow-up data was addressed by replicating that participant's last observed score on that measure. Follow-up data were not available for control group participants who had begun treatment by that time.

### Post-Treatment (11-Week) Outcomes

#### Primary Outcomes

Univariate ANCOVAs on post-treatment PHQ-9 and BDI-II scores, controlling for pre-treatment scores (see Table 2), revealed significant effects for PHQ-9 (*F_2, 123_* = 29.72, p<0.001) and BDI-II (*F_2, 123_* = 21.72, p<0.001) scores. Post-hoc pairwise comparisons revealed no difference on either measure between treatment groups, but significant differences between the treatment groups and the control group (p<0.001).

**Table 2:** Results of outcome measures: Means, standard deviations, confidence intervals and effect sizes (Cohen's *d*) for each group (intention to treat; last observation carried forward).

	Group		Pre	Post	Pre-Post	Effect Sizes*			Follow-Up	Pre-Follow-Up	Effect Sizes*	
Outcome Measure		n	Mean (SD)	Mean (SD)	Mean Difference (95% CI)	Within Group	TA vs. CA	vs. Control	Mean (SD)	Mean Difference (95% CI)	Within	TA vs. CA
PHQ-9	TA	41	14.20 (4.20)	7.59 (4.04)	6.61 (4.95–8.27)	1.60	0.07	1.27	6.49 (3.94)	7.71 (6.13–9.28)	1.89	0.46
	CA	46	14.15 (4.39)	7.30 (4.48)	6.85 (5.40–8.29)	1.54		1.31	8.67 (5.42)	5.48 (3.75–7.20)	1.11	
	Control	40	13.35 (4.62)	12.98 (4.44)	0.38 (−0.91–1.66)	0.08						
BDI-II	TA	41	27.15 (9.96)	15.29 (9.81)	11.85 (7.85–15.85)	1.20	0.07	1.09	11.66 (9.58)	15.49 (11.62–19.36)	1.59	0.40
	CA	46	28.96 (11.51)	14.59 (11.12)	14.36 (10.62–18.11)	1.27		1.09	16.22 (13.16)	12.74 (8.83–16.65)	1.03	
	Control	40	26.33 (10.46)	26.15 (10.14)	0.18 (−2.12–2.77)	0.02						
K-10	TA	41	28.32 (6.39)	21.90 (7.01)	6.41 (3.85–8.98)	0.96	0.12	0.71	18.63 (7.64)	9.68 (6.71–12.66)	1.38	0.45
	CA	46	29.07 (7.37)	21.07 (6.46)	8.00 (5.85–10.15)	1.15		0.86	22.04 (7.39)	7.02 (4.79–9.25)	0.95	
	Control	40	28.30 (7.29)	26.98 (7.29)	1.33 (−0.52–3.17)	0.18						
SDS	TA	41	18.17 (6.69)	10.51 (8.03)	7.66 (4.87–10.45)	1.04	0.06	0.86	8.80 (7.56)	9.37 (6.64–12.09)	1.31	0.36
	CA	46	18.22 (6.83)	11.00 (7.60)	7.22 (4.90–9.54)	1.00		0.80	11.65 (8.29)	6.56 (4.31–8.82)	0.87	
	Control	40	16.95 (7.05)	16.38 (5.65)	0.58 (−1.37–2.52)	0.09						

Pre: Pre treatment, Post: Post-treatment; Follow-up: 4 month follow-up; PHQ-9: Patient Health Questionnaire 9-Item; BDI-II: Beck Depression Inventory Second Edition; K-10: Kessler 10-Item; SDS: Sheehan Disability Scale; TA: Technician assisted; CA: Clinician assisted. CI: Confidence Interval.

Note. *All effect sizes are absolute values.

#### Secondary Outcomes

Univariate ANCOVAs conducted on the K-10 and SDS post-treatment scores, while controlling for pre-treatment scores, revealed significant effects for K-10 (*F_2, 124_* = 13.06, p<0.001) and SDS (*F_2, 124_* = 63.87, p<0.001) scores. Post-hoc pairwise comparisons revealed no difference on either measure between treatment groups, but significant differences between the treatment groups and the control group (p<0.001).

#### Effect Sizes

Pre to post-treatment within-group effect sizes on the BDI-II were 1.27 and 1.20 for the clinician and technician-assisted groups, respectively, and on the PHQ-9 were 1.54 and 1.60, respectively (Table 2). The between-group effect size for the treatment groups on the BDI-II was 0.07 (CI95% = −0.35 to 0.49) and on the PHQ-9 was 0.07 (CI95% = −0.36 to 0.49). Large (>0.80) within-group effect sizes (ESs) were found for both treatment groups on the K-10 and SDS. Large ESs between each treatment group and the control group were found for all measures.

#### Clinical Significance: Remission, Recovery, and Reliable Clinical Change

At pre-treatment 37/41 (90%) of TA group, 41/46 (89%) of CA group, and 31/40 (78%) of control group participants had a PHQ-9 score of 10 or more, indicating a persisting diagnosis of depression. At post-treatment (using the ITT and LOCF design) 11/41 (27%) of TA group, 18/46 (39%) of CA group, and 31/40 (78%) of control group participants continued to have a PHQ-9 score above 9. Based on the criteria for recovery (a reduction of pre-treatment PHQ-9 scores of at least 50%) at post-treatment, 61% of TA group, 43% of CA group and 3% of control group participants were classified as recovered. Based on the criteria for reliable clinical change (statistically reliable change and a reduction of pre-treatment BDI-II scores of at least 10 points), 56% of TA group, 61% of CA group and 8% of control group participants were classified as having achieved reliable clinical change at post-treatment.

#### Treatment Satisfaction

Chi-squared tests and one-way ANOVAs failed to reveal any differences between treatment groups' ratings of satisfaction with the program with respect to: Overall satisfaction (p = .13); quality of the treatment lessons (p = .82); and quality of the support they received from the technician or clinician (p = .40). Overall, treatment group participants reported an acceptable level of satisfaction with the overall program, with 67/77 (87%) reporting being either *very satisfied* or *mostly satisfied*, 10/77 (13%) *neutral/somewhat dissatisfied,* and 0% reporting *very dissatisfied*. Most responding participants (90%) rated the quality of the treatment modules as *excellent* or *good*; 81% rated the quality of Internet correspondence with the clinician or technician as *excellent* or *good*, 14% rated it as *satisfactory,* and 4% as *unsatisfactory*.

When asked to provide a rating from 1 to 10, where 10 indicates a high level of agreement, the average participant rated the treatment as logical (8/10); they reported feeling confident that the treatment would be successful at teaching them techniques for managing their symptoms (8/10); and they reported a high level of confidence in recommending this treatment to a friend with depression (8/10). No between treatment group differences were found on these items.

#### Time/Contact Events Per Participant

One-way ANOVAs did not reveal any difference in the total number of contacts (telephone calls and emails) provided during the 8 week program to each participant in the TA (mean, SD; 36.9, 5.3) and CA groups (34.7, 6.3) (p = .10). One-way ANOVAs also failed to reveal any difference in the total time spent by the technician (mean, SD, 61.0 mins, 9.8) or clinician (60.5, 19.0) with each participant during the program (p = .88). These time estimates included time required monitoring individual progress, reading and responding to emails, discussing cases with the clinician, and attending weekly supervision sessions. Conducting this research added approximately 5 hours per week to the technician and the clinician's existing workload. The technician reported that most TA group participants were satisfied during treatment but four (10%) participants were discussed with the clinician because of concerns about their progress. The clinician intervened in two of these cases, withdrawing them from the program with their consent, and referring them to other services.

#### Time/Contact Events Per Participant and Change Scores

No significant correlations were found between change scores on the primary outcome measures and total time or total contact events per participant (p range = 0.48–0.97).

### Follow-Up (4-Month) Outcomes

#### Primary Outcomes

Univariate ANCOVAs on follow-up PHQ-9 and BDI-II scores, controlling for pre-treatment scores (see Table 2), revealed a significant difference between the CA and TA groups on the PHQ-9 (*F_1, 84_* = 4.94, p<0.05) in favour of the TA group, but no difference between groups on the BDI-II (p = .10). Paired samples *t*-tests for each intervention group revealed that the TA group made significant improvements between post-treatment and follow-up assessments on the PHQ-9 (t(40) = 2.48, p<0.05) and BDI-II (t(40) = 3.29, p<0.05). They also revealed that the CA group deteriorated between post-treatment and follow-up assessments on the PHQ-9 (t(45) = 2.19, p<0.05) but had not changed on the BDI-II (p = 0.18).

#### Secondary Outcomes

Univariate ANCOVAs conducted on the K-10, and SDS follow-up scores, while controlling for pre-treatment scores, revealed a significant difference between the CA and TA groups on the K-10 (*F_1, 84_* = 4.18, p<0.05), but not on the SDS.

Paired samples *t*-tests for each intervention group revealed that the TA group made significant improvements between post-treatment and follow-up assessments on the K-10 (t(40) = 2.59, p<0.05) and SDS (t(40) = 2.04, p<0.05), with no significant changes in CA group scores.

**Effect Sizes.** Pre to follow-up within-group effect sizes (Table 2) on the BDI-II were 1.03 and 1.59 for the clinician and technician-assisted groups, respectively, and 1.11 and 1.89 on the PHQ-9, respectively. The between-group effect size for the treatment groups on the BDI-II was 0.39 (CI95% = −0.03 to 0.82) and on the PHQ-9 was 0.46 (CI95% = 0.03 to 0.88). Large (>0.80) within-group effect sizes (ESs) were found for both treatment groups on the K-10 and SDS.

#### Clinical Significance: Remission, Recovery, and Reliable Clinical Change

At follow-up (using the intention-to-treat and LOCF design) 9/41 (22%) of TA group and 18/46 (39%) of CA group participants continued to have a PHQ-9 score above 9. Based on the criteria for recovery (a reduction of pre-treatment PHQ-9 scores of at least 50%) at follow-up 66% of TA group and 43% of CA group participants were classified as recovered. Based on the criteria for reliable clinical change (statistically reliable change and a reduction of pre-treatment BDI-II scores of at least 10 points) 59% of TA group and 59% of CA group participants were classified as having achieved reliable clinical change at follow-up.

## Discussion

This trial examined the relative efficacy and acceptability of technician- vs. clinician-assisted Internet-based CBT for depression. At intake all participants met DSM-IV diagnosis of major depressive episode, and the majority reported a history of multiple depressive episodes. In addition to having access to the components of the Sadness program, CA group participants had weekly email or telephone contact with a clinician and access to an online discussion forum. The clinician actively engaged in treatment with CA group participants. TA group participants also had access to the Sadness program and weekly email or telephone contact with the technician, but did not have access to an online forum. The technician provided support and encouragement, but did not provide clinical advice, and was instructed to refer clinical questions or concerns to the clinician.

At post-treatment outcomes for both treatment groups were superior to the control group and there were no differences between the two treatment groups in either clinical outcomes or acceptability. ESs within each treatment group on both depression measures was equal to or greater than to 1.2 indicating that the treatment effect was considerable. At post-treatment approximately 50% of participants in the treatment groups were classified as recovered and/or had achieved reliable clinical change, providing further evidence of clinical efficacy. Satisfaction with treatment was high, even though the amount of contact time per participant was approximately one hour. The total contact time required for each group of more than 40 participants was estimated at approximately 5 hours per week of clinician or technician time. The automated email system facilitated the efficiency of contact with participants and during treatment participants received more than 30 contacts, prompts, and reminders.

At follow-up this pattern of results was generally stable, although the technician-assisted group continued to make improvements. At follow-up, the technician-assisted had significantly lower PHQ-9 and K-10 scores than the clinician-assisted group. At follow-up the between-group effect size for the technician-assisted group (1.89) on the PHQ-9 was considerably larger than for the clinician-assisted group (1.11).

### Generalizability

These results support the study hypotheses and are consistent with recent findings indicating that less than one hour of therapist support during I-CBT for depression can produce excellent clinical outcomes [11]. The present results also provide evidence that a technician, when supervised by a clinician, can produce results similar to those obtained by mental health professionals in the treatment of depression. Similar outcomes have been reported in I-CBT programs for treating social phobia [28], [29] and generalized anxiety disorder [16], providing evidence that, when supervised by a clinician, I-CBT programs may be effectively and safely administered by a non-clinician. The technician in the present study was able to “step-up” participants to the clinician, but did so with only 10% of participants. This indicates that the majority found the intervention by the technician sufficient for their needs, and demonstrates a potential model for integrating clinician and technician support during iCBT programs. The magnitude of clinical effects observed in this study are consistent with those observed in previous evaluations of clinician-assisted Internet-based treatment of depression [9]–[13]. These results are also comparable to those observed in face-to-face treatment [48], but considerably larger than those observed in self-guided Internet-based treatment programs for depression [49], [50].

An unexpected finding was that the technician-assisted group continued to make improvements between post-treatment and follow-up, while the clinician-guided group did not improve and deteriorated on one of the primary outcome measures. It is difficult to account for these findings as pre-treatment expectancies and post-treatment satisfaction did not differ between intervention groups, and no between-group differences were found in the total number of contacts with participants or in the time spent on each participant. Replication of this research design and detailed analysis of the messages sent by the clinician and technician will be conducted to explore the reliability of this finding and possible reasons for differences.

### Limitations

The relatively small sample size is one limitation of this study. Other possible limitations include that the TA group had fewer males than the other groups, and that the CA group had access to more resources than the TA group. However, there is no indication that these issues affected results. A fourth limitation is the self-selecting nature of the sample. Those volunteering may well be inherently motivated, but this does not challenge the internal validity of the results. Another important potential limitation is the use of a delayed treatment control group rather than an attention-control placebo. This choice was grounded in concerns about the impact of raised expectations of symptom resolution in depressed participants placed in a placebo, attention-control condition, and the difficulty contacting Internet participants should additional help have been required.

### Conclusions

This randomized controlled trial found no difference between a clinician- and technician-assisted Internet-based treatment program for depression at post-treatment. Both conditions resulted in large effect sizes, clinically significant improvements, and high levels of acceptability, while a delayed treatment control group did not improve. The technician-assisted group continued to improve at the 4 month follow-up, while the clinician-assisted group did not. These results demonstrate the potential of a new model of providing evidence-based treatment for depression. The question is not whether to accept such an innovative model of service delivery, but how to do so in an ethical, competent, safe, and cost-effective way, while also maintaining excellent clinical standards.

## Supporting Information

Checklist S1 CONSORT Checklist
(0.19 MB DOC)

Protocol S1 Trial Protocol
(0.41 MB PDF)
