# Supplementary material for: Internet Treatment for Depression: A Randomized Controlled Trial Comparing Clinician vs. Technician Assistance
Source: PLoS One. 2010 Jun 8;5(6):e10939. doi: 10.1371/journal.pone.0010939 (PMC2882336; doi:10.1371/journal.pone.0010939)
Supplement: Protocol S1 — Trial Protocol (0.41 MB PDF) [file pone.0010939.s002.pdf]

NSW Health On−line Forms
National Ethics Application Form

Short title and version number: (maximum 70 characters)
Distance treatment III: RCT in social phobia, panic and depression

Name/ID of HREC reviewing the research project:
St Vincent's Hospital Human Research Ethics Committee (EC00140)

HREC Application Reference Number: 08/SVH/36

Submission date: 02/04/2008

1. TITLE AND SUMMARY OF PROJECT

1. What is the formal title of this research proposal?

Full title: Distance treatment III: Randomised controlled trial in social phobia, panic and depression

Key words:

2. Description of the project in plain language

Give a concise and simple description (not more than 400 words), in plain language, of the aims of this project, the proposal research design and the methods to be used to achieve those aims.

Many people with social phobia, panic disorder or depressive disorder do not access treatment. Internet based therapies exist but adherence is poor without clinician guidance. We have modified existing patient education internet programs used by GPs as the basis for clinician guided computerised cognitive behaviour therapy programs (the interventions) for adults with social phobia, panic disorder or with depression.

This phase 2b study will recruit 105 people with panic disorder, 195 people with depression, and 105 people with social phobia and randomly allocate them to clinician assisted computerised cognitive behaviour therapy (CaCCBt) or computerised cognitive behaviour therapy (CCBT) or to remaining on a waitlist with the prospect of active treatment after 13 weeks. We will measure changes in symptom level from the beginning of the the 6−10 week course of treatment and one week after the conclusion of treatment. Measurement in the control group will be matched to this. We will also measure adherence to the lessons, homework and forum; satisfaction with the clinician's input and satisfaction with the mode of treatment generally.

3. Type of Research

Tick as many of the following 'types of research' as apply to this project. Your answers will assist HRECs in considering your proposal. A tick in some of these boxes will generate additional questions relevant to your proposal (mainly because the National Statement requires additional ethical matters to be considered), which will appear in Section 4 of NEAF.

The project involves:

- ☐ Qualitative research
- ☐ Research on workplace practices or possibly impacting on workplace relationships
- ☐ Research conducted overseas involving participants
- ☐ Research involving deception of participants, concealment or covert observation
- ☐ Epidemiological research
- ☐ Administration of a drug for research but is not clinical research

- [x] Clinical research (excluding those under the CTN/CTX schemes)
- [ ] Clinical trial under CTN/CTX scheme
- [ ] Research involving ionising radiation
- [ ] Research involving the use of embryos and/or gametes
- [ ] Genetic testing/research
- [ ] Research involving the collection and / or use of human samples
- [ ] Research involving assisted reproductive technologies (ART)
- [ ] None of the above

4. Research participants

The National Statement requires additional information to be provided to an HREC where research participants are certain or likely to include any of the categories of people listed in this question. HRECs need to know whether you intend to include or to exclude any of these categories. Answer this question by
(a) selecting any of those categories that are targeted or likely to be included as participants in this research project,
(b) selecting any other of these categories that will be excluded from participation, and
(c) selecting any other of these categories who may be adversely affected by this research.

Where you select a category for inclusion, you will be required to answer additional questions later in the form.

Where any of the following participant populations may be involved, the National Statement requires additional information to be provided to the HREC. Tick as many of the following 'types of research participants' as apply to this project. If none apply please indicate this below. A tick in some of these boxes will require you to answer additional questions later in the form.

**The participants who may be involved in this research are:**<br><br>***If you select column (a) or (b), column (c) will not apply.***	**a) Intended or targeted**	**b) Probable coincidental recruitment**	**c) Design specifically excludes**	**d) Research has potential to adversely affect this population**
People whose primary language is other than English (LOTE)	☐	☑	☐	☐
Children and/or young people (ie. <18 years)	☐	☐	☑	☐
People with an intellectual or mental impairment	☐	☐	☑	☐
People highly dependent on medical care	☐	☐	☐	☐
People in existing dependent or unequal relationships with any member of the research team, the researcher(s), and/or the person undertaking the recruitment/consent process (eg. student/teacher; employee/employer; warden/prisoner; officer, enlisted soldier; patient/doctor)	☐	☐	☐	☐
People who belong to a collectivity	☐	☐	☐	☐
Aboriginal and/or Torres Strait Islander peoples	☐	☐	☐	☐

5. Research techniques

The research techniques to be used in this project include: *(You must tick at least one. Tick as many as apply)*

- [ ] Observation of non−identified people in public places
- [ ] Covert observation of identifiable people in non−public places
- [x] Interviews − telephone
- [ ] Interviews − face to face
- [ ] Documentary/records analysis
- [ ] Focus groups
- [ ] Data linkage
- [ ] Physical activities / exercises / tests
- [ ] Taping − audio / video
- [ ] Biomedical / clinical interventions, tests, samples
- [ ] Use of complementary or alternative medicine, or a natural therapy
- [ ] Photos
- [ ] Use of gene therapy
- [x] Survey instrument / questionnaire / diary
- [ ] Use of a medical device
- [x] Internet / web based research
- [ ] Computer based tests
- [ ] Other techniques

2. RESEARCHERS

1. Applicant

Provide the following information for the person making this application to the HREC.

	Title:	Forename/Initials:	Surname:
	Professor	John Gavin	Andrews

Mailing Address:	299 Forbes St
Suburb/Town:	Darlinghurst
State:	NSW
Postcode:	2010
Country:	Australia
Organisation:	UNSW at St Vincent's Hospital, Sydney
Department*:	Clinical Research Unit for Anxiety and Depression
Position:	Director
E−mail:	gavina@unsw.edu.au
Phone (BH):	8382 1726
Phone (AH)*:	
Mobile*:	
Pager*:	
Fax:	8382 1721

2. Principal researcher(s)

Principal researcher 1

	Title:	Forename/Initials:	Surname:
	Professor	John Gavin	Andrews

Mailing Address:	299 Forbes St
Suburb/Town:	Darlinghurst
State:	NSW
Postcode:	2010
Country:	Australia
Organisation:	UNSW at St Vincent's Hospital, Sydney
Department*:	Clinical Research Unit for Anxiety and Depression
Position:	Director
E−mail:	gavina@unsw.edu.au
Phone (BH):	8382 1726
Phone (AH)*:	
Mobile*:	
Pager*:	
Fax:	8382 1721

Describe the qualifications, expertise and experience of the principal researcher relevant to this project.

Qualifications:	MD FRCPsych FRANZCP
Expertise:	Authored numerous books and journal articles on anxiety and depression treatment
Experience:	Director of anxiety disorders clinic for 30 years
Name the site(s) for which this principal researcher is responsible:	St Vincent's Hospital, Sydney
Describe the role of the principal researcher in this project:	Director
Is the principal researcher a student?	◯ Yes ◉ No

Principal researcher 2

	Title:	Forename/Initials:	Surname:
	Dr	Nickolai	Titov

Mailing Address:	299 Forbes St
Suburb/Town:	Darlinghurst
State:	NSW
Postcode:	2010
Country:	Australia
Organisation:	UNSW at St Vincent's Hospital, Sydney
Department*:	
Position:	Senior Lecturer
E–mail:	nickt@unsw.edu.au
Phone (BH):	8382 1732
Phone (AH)*:	
Mobile*:	
Pager*:	
Fax:	8382 1721

Describe the qualifications, expertise and experience of the principal researcher relevant to this project.

Qualifications:	PhD
Expertise:	Clinical psychologist with special interest in internet therapy
Experience:	Developed the model for distance treatment using clinician assisted CBT
Name the site(s) for which this principal researcher is responsible:	St Vincent's Hospital Sydney
Describe the role of the principal researcher in this project:	Advise the associate investigators
Is the principal researcher a student?	◯ Yes ◉ No

3. Associate Researchers

How many known associate researchers are there? 3

Do you intend to employ other associate researchers? ◯ Yes ◉ No

Associate Researcher 1

	Title:	Forename/Initials:	Surname:
	Ms	Sarah	Perini

Mailing Address: 299 Forbes St

Suburb/Town: Darlinghurst
State: NSW
Postcode: 2010
Country: Australia
Organisation: St Vincent's Hospital, Sydney
Department*:
Position: Clinical Psychologist
E–mail: sperini@stvincents.com.au
Phone (BH): 8382 1729
Phone (AH)*:
Mobile*:
Pager*:
Fax: 8382 1721

Describe the qualifications, expertise and experience of the associate researcher relevant to this project.

Qualifications:	M Clinical Psychol
Expertise:	Experienced clinician with expertise in the cognitive behaviour therapy of depression
Experience:	Four years of clinical experience in this work
Description of the role of the associate researcher in this project:	Conduct the treatment of people with depression
Name the site at which the associate researcher has responsibility:	St Vincent's Hospital

Is the researcher a student? ◯ Yes ◉ No

Associate Researcher 2

	Title:	Forename/Initials:	Surname:
	Dr	Edward	Wims

Mailing Address: 299 Forbes St

Suburb/Town: Darlinghurst
State: NSW
Postcode: 2010

Country: Australia
Organisation: St Vincent's Hospital
Department*:
Position: Senior Registrar
E–mail: ewims@stvincents.com.au
Phone (BH): 8382 1747
Phone (AH)*:
Mobile*:
Pager*:
Fax: 8382 1721

Describe the qualifications, expertise and experience of the associate researcher relevant to this project.

Qualifications:	MB Bch BAO, MRCPsych
Expertise:	Trained in CBT for people with panic disorder
Experience:	Two years experience in this treatment
Description of the role of the associate researcher in this project:	Conduct the treatment of people with panic disorder
Name the site at which the associate researcher has responsibility:	St Vincent's Hospital

Is the researcher a student? ◯ Yes ◉ No

Associate Researcher 3

Title:	Forename/Initials:	Surname:
Dr	Alison	Mahoney

Mailing Address: 299 Forbes St

Suburb/Town: Darlinghurst
State: NSW
Postcode: 2010
Country: Australia
Organisation: St Vincent's Hospital
Department*:
Position: Cinical Psychologist
E–mail: amahoney@stvincents.com.au
Phone (BH): 8382 1720
Phone (AH)*:
Mobile*:
Pager*:
Fax: 8382 1721

Describe the qualifications, expertise and experience of the associate researcher relevant to this project.

Qualifications:	PsyD
Expertise:	Clinical psychologist with experience in cognitive behaviour therapy for anxiety disorders
Experience:	Two years experience treating people with social phobia
Description of the role of the associate researcher in this project:	Treating people with social phobia in the CaCCBT condition
Name the site at which the associate researcher has responsibility:	St Vincent's Hospital
Is the researcher a student?	○ Yes ◉ No

4. Other personnel relevant to the research project

a. How many known other people will play a specified role in the conduct of this research project?

1

b. Describe the role, and expertise where relevant (e.g. counsellor), of these other personnel.

Research assistant, B Sc (Hons in Psychology)

c. Is it intended that other people, not yet known, will play a specified role in the conduct of this research project?

○ Yes ◉ No

5. Certification of researchers

a. Are there any relevant certification, accreditation or credentialing requirements relevant to the conduct of this research?

○ Yes ◉ No

b. Describe the certification, accreditation or credentialing requirements.

c. Specify and advise whether the principal researcher or any of the associate researchers have been so certified and/or accredited or credentialed.

6. Training of researchers

a. Do the researchers or others involved in any aspect of this research project require any additional training in order to undertake this research?

◉ Yes ○ No

b. What is this training?

A manual based on shyness protocols has been written and Perini, Wims, and Mahoney will be familiarized with it.

c. How and by whom will the training be provided?

Dr Titov

d. How will the outcome of the training be evaluated?

Demonstrated competence

3. PROJECT

Duration and location

1. In how many Australian sites, or site types, will the research be conducted?

1

2. In how many overseas sites, or site types, will the research be conducted?

0

3. Provide the following information for each site or site type (Australian and overseas, if applicable) at which the research is to be conducted

1

Site / Site Type Name:	St Vincent's Hospital
Site / Site Type Location:	Victoria St Darlinghurst

4. Provide the start and finish dates for the whole of the study including data analysis

Start date:	10/04/2008 *(dd/mm/yyyy)*
End date:	31/12/2009 *(dd/mm/yyyy)*

5. Are there any time-critical aspects of the research project of which an HREC should be aware?

○ Yes ◉ No

Research plan

6. Describe the theoretical, empirical and/or conceptual basis, and background evidence, for the research proposal, eg. previous studies, anecdotal evidence, review of literature, prior observation, laboratory or animal studies.

In the Australian National Survey of Mental Health and Well Being only 60% of people with depression reported seeing a physician and approximately only half got adequate treatment. The picture for panic disorder was worse, only 40% saw a physician and again approximately only half got adequate treatment. The picture for social phobia was terrible, only 20% saw a physician and again only half probably got adequate treatment (Andrews et al, 2004). The reason for the shortfall is well known (Andrews et al, 2001) being a mixture of societal, attitudinal and diagnostic variables. Making treatment more freely available in areas where expert treatment is not available, or to people who are unable to take time off work to access treatment is one logical step. This series of studies aims to achieve this.

Social phobia, panic disorder and depression respond to cognitive behaviour therapy (CBT) and to medication with SSRIs (Nathan & Gorman, 2007). CBT for these disorders can be delivered over the internet, but one difficulty with computerised CBT (CCBT) is that adherence is usually extremely poor in the absence of clinician input (Titov, 2007). We recently completed a randomised controlled trial of clinician assisted computerised CBT (CaCCBT) for the distance treatment of people with social phobia (Titov, in press). Adherence was 80%. People in the intervention groups were as severe as people seen in the specialised anxiety disorders clinic at St Vincent's Hospital and yet made the same level of improvement as did people receiving face to face treatment (ES respectively 0.8 clinic, 1.2 CaCBT) but the latter group only required one quarter as much clinician time. This finding has been replicated (Titov, Andrews and Schwencke, in press).

We have applied for ethics clearance (Distance Treatment I)for a Phase 1 feasibility study of clinician assisted web based CBT (CaCCBT) for people with panic disorder and for people with depression. When that study is complete in June 08 we would seek to extend that finding by conducting a Phase 2a study (Distance treatment II) comparing CaCCBT with wait list in panic and depression. This Distance treatment III, Phase 2b study is next. It compares CaCCBT with CCBT and waitlist. The social phobia component can begin before the other trials are complete because the Phase 2a comparison with wait list in social phobia is complete.

7. State the aims of the research and the research question and/or hypotheses, where appropriate.

To explore the outcomes of the clinician assisted CBT programs for people with social phobia, panic disorder or depression compared to the status of people who receive CCBT or who remain on the wait list using a RCT design.

To further determine the feasibility of these treatments in terms of acceptability to patients and practicality for clinicians.

8. Describe how the research design and the methods to be used will enable the research aims to be achieved.

The Phase 1 question was whether the Clinician assisted computerised CBT is a potentially valuable treatment in terms of cost (clinician time) and patient benefit. The likelihood of harm was judged to be very low and from the Phase 2a trials with social phobia the possibility of benefit was high given such an experienced team. If both these predictions are confirmed we will proceed to the present study.

Study design: a CONSORT compliant, registered RCT of the intervention (CaCCBT) versus CCBT vs waitlist control group. This will demonstrate whether the benefit from the intervention is superior to natural remission and placebo response. Because of low adherence to CCBT we think that it is a proxy for a placebo treatment. As in any RCT accurate patient selection, good randomization, reliable and valid outcome measures, and low drop out rates are critical. Analyses based on completers and on intention to treat will be performed.

Patient selection: Recruiting for the Shyness study left us with nearly 100 people whose depression was too significant to allow them to be included in that study. We will contact those people and offer them treatment in the present study. We will contact people on the waiting list with social phobia. On the 23rd April we have a radio interview with "Drivetime" with Richard Glover to report the results of the shyness study. We will use that opportunity to publicise the panic, depression and social phobia study.

Inclusion/exclusion criteria: age over 18, self identified as suffering from social phobia or panic or depression and have questionnaire scores and results of telephone diagnostic interview consistent with this; no history of psychosis or current alcohol and drug dependence, not currently suicidal; have access to phone and computer with printer. Prepared to provide name, phone number and address and to be registered as a patient of St Vincent's Hospital, Sydney, and to provide the name and address of a local general practitioner. Provide written informed consent.

Study assessments:
Triage: PHQ9 (9 items); GAD7 (7 items); self identification of principal complaint.
Pre−post measures: K10; WHODAS2; Patient satisfaction scale (appended)for all groups. Center for Epidemological Studies Depression Scale (CES−Depression scale), Depression Anxiety and Stress Scale (DASS) for depression group; Agoraphobic Cognitions Questionnaire (ACQ) and Body Sensations Questionnaire (BSQ) for the panic group; Social Phobia Scale (SPS) and Social Interaction Anxiety Scale (SIAS) for the social phobia group.

Power: We would expect pre−post improvement of ES 0.5−0.8. We also expect the CaCCBT group to improve more than the CCBT group and waitlist group by and ES of 0.3 (for the depression group) and 0.6 (for panic and social phobia). Sample size is powered to have an 80% chance of detecting differences at p<.05.

Research significance

9. What is the value of answering the research question and conducting the project?

The Phase 1 study will test whether the intervention is potentially efficacious. The Phase 2a study will test whether it is superior to natural history in these disorders. This knowledge will justify continued evaluative research. The Phase 2b study comparing the intervention (CaCBT) with internet based CBT without clinician assistance and with a wait list control. True placebo treatments are difficult but the comparison with CCBT alone will provide the expectation that people are in treatment and hence control for the natural history and for the placebo effect.

Peer review

10. Has the research proposal, including design, methodology and evaluation undergone, or will it undergo, a peer review process?

(•) Yes ( ) No

Provide details of the review and the outcome. A copy of the letter / notification, where available, should be attached to this application.

It was approved at the weekly research meeting of the Clinical Research Unit for Anxiety and Depression.

The following questions sets (Q 11 – Q 40) relate to the collection, use and disclosure of information for research purposes. In answering these questions please ensure that you address all issues relevant to the type of project and type of participants that will be involved in your research project. Refer for guidance to relevant chapters of the National Statement, NEAF Guidance and other NHMRC guidelines as appropriate.

Source and description of information about participants

11. Indicate the source of the information about participants which will be used in this research project.

- [x] Information will be collected directly from the participant.
- [ ] Information will be collected from another person about the participant
- [ ] Information will be collected by accessing a record or an information database held by an organisation other than your organisation.
- [ ] Information will be used which you or your organisation collected previously for a purpose other than this research project.

11a. Describe the information that will be collected directly from participants. Be specific where appropriate.

Name and address/phone number, age, gender, and demographic details.
Self identified principal disorder, confirmed by telephone diagnostic interview with the Mini International Neuropsychiatric Interview.
Information about psychotic symptoms and substance abuse for purposes of exclusion.
Scores on the relevant social phobia, panic and depression scales and on the general measures of stress and of disability.

The information collected by the research team about participants will be in the following form(s). Tick more than one box if applicable.

- [x] Identified
- [ ] Potentially identifiable (coded)
- [ ] De−identified

Give reasons why it is necessary to collect information in identified or potentially identifiable (coded) form.

They will be patients of the Hospital and this information is collected as part of their distance treatment and the details will be recorded in their hospital medical record. Hospital records are usually identified.
Furthermore, the clinician will need to collect identified information from the patient in order to monitor their progress as part of good clinical practice.

Will consent be sought from participants (or for participants from persons with legal authority) for the collection and use of information about them?

(•) Yes ( ) No

Use of information about participants

15. Describe how information collected about participants will be used in this project.

Scores will be used to determine suitability for treatment and then to calculate their improvement with treatment. This knowledge will enable the clinician to debrief each patient appropriately with advice as to a prudent next step in treatment if required when the trial is over.

16. Will any of the information used by the research team be in identified or potentially identifiable (coded) form?

(•) Yes ( ) No

Give reasons why it is necessary to use information in identified or potentially identifiable (coded) form.

This is an evaluation of a program of clinical treatment. The responsible clinician will know this information as part of clinical responsibility, the other members of the research team will only see deidentified data, ie. without name or address or phone number. The overall results, but not any individual data, will be used to inform the next stage of the research.

Indicate whichever of the following applies to this project:

- [x] Information collected for, used in, or generated by, this project will not be used for any other purpose.
- [ ] Information collected for, used in, or generated by, this project will/may be used for another purpose by the researcher for which ethical approval will be sought.
- [ ] Information collected for, used in, or generated by, this project is intended to be used for establishing a database/data collection/register for future use by the researcher for which ethical approval will be sought.
- [ ] Information collected for, used in, or generated by, this project will/may be made available to a third party for a subsequent use for which ethical approval will be sought.

17. List ALL research personnel and others who, for the purposes of this research, will have authority to use or have access to the information and describe the nature of the use or access. Examples of others are: student supervisors, research monitors, pharmaceutical company monitors.

Only the responsible clinician will see identified data. De−identified data will be seen by all five investigators and the research assistant in the analysis and write up phases. In the social phobia RCT patients gave permission for some of their deidentified postings on the forum to be displayed to help others. We presume that this will occur in this trial across all three disorders.

Storage of information about participants during and after completion of the project

18. In what formats will the information be stored during the research project? (eg. paper copy, computer file on floppy disk or CD, audio tape, videotape, film)

Identified data will be stored securely on a password protected computer,deidentified data will be stored as a computer file with back up.
We will also create a paper medical record that will contain name, address, phone number and email address plus summary scores on assessment scales before and after treatment. People in the sadness program will be asked for a GPs name and address.

19. Specify the measures to be taken to ensure the security of information from misuse, loss, or unauthorised access while stored during the research project? (eg. will identifiers be removed and at what stage? Will the information be physically stored in a locked cabinet?)

Identified and deidentified data will be password protected from unauthorised access. Identifiers will be removed when the scores are transferred from the individual patient records to the data base to form the deidentified data.
Identified data (medical records) will become the property of the Hospital.

20. In what formats will the information be stored after project completion? (eg. paper copy, computer file on floppy disk or CD, audio tape, videotape, film)

Deidentified data will be stored as a computer file with back up. Identified medical record data in paper and kept at the hospital.

21. Specify the measures to be taken to ensure the security of information from misuse, loss, or unauthorised access while stored after project completion (eg. will identifiers be removed and at what stage? Will the information be physically stored in a locked cabinet?)

Numerical codes will contain no links to the identity of the person.

22. The information which will be stored at the completion of this project is of the following type(s). Tick more than one box if applicable.

- [x] Identified
- [ ] Potentially identifiable (coded)
- [x] De−identified

Give reasons why it is necessary to store information in identifiable or potentially identifiable (coded) form.

The records form part of the patients medical record, hospital records are identifiable.

23. For how long will the information be stored after the completion of the project and why has this period been chosen?

For 7 years

24. What arrangements are in place with regard to the storage of the information collected for, used in, or generated by this project in the event that the principal researcher ceases to be engaged at the current organisation?

His successor will take charge

Ownership of the information collected during the research project and resulting from the research project

25. Who owns the information collected for the research project?

St Vincent's Hospital

26. Who is understood to own the information resulting from the research, eg. the final report or published form of the results?

The five investigators

27. Does the owner of the information or any other party have any right to impose limitations or conditions on the publication of the results of this project?

○ Yes ◉ No

Disposal of the information

28. Will the information collected for, used in, or generated by this project be disposed of at some stage?

◉ Yes ○ No

At what stage will the information be disposed?

7 years.

How will information, in all forms, be disposed?

Paper records will be disposed of via medical record clearance; the computer database will be erased after 7 years.

Reporting individual results to participants and others

29. Is it intended that results of the research that relate to a specific participant be reported to that participant?

◉ Yes ○ No

Specify in what form the results will be reported to participants:

The treating clinician will conduct an email interchange with that participant

How will the results be communicated to participants? eg telephone call, individual letter, copy of publication, consultation with a medical practitioner or other

Email

Who will be responsible for communicating the project results to participants?

The treating clinician

30. Is the research likely to produce information of personal significance to individual participants?

◉ Yes ○ No

31. Will individual participant's results be recorded with their personal records?

◉ Yes ○ No

32. Is it intended that results that relate to a specific participant be reported to anyone other than that participant?

◉ Yes ○ No

To whom will the results be reported other than the participant?

Participants will be required to nominate a GP to whom we can report. We will do this should there be reasons for concern, i.e. sustained high depression scores (PHQ−9 >20)

Explain why the results will be reported to a person other than the participant?

We will have permission to communicate with their own doctor.

Will the participant be told that their results will be reported to another person?

(•) Yes ( ) No

33. Is the research likely to reveal a significant risk to the health or well being of persons other than the participant, eg family members, colleagues

( ) Yes (•) No

Dissemination of Final Results

34. How is it intended to disseminate the results of the research? eg report, publication, thesis

In journal articles and at conferences

35. Will the confidentiality of participants and their data be protected in the dissemination of research results?

(•) Yes ( ) No

Explain how confidentiality of participants and their data will be protected in the dissemination of research results:

Only deidentified data will be available to the researchers when they analyse the data, only grouped data will be reported

36. Is there a risk that the dissemination of results could cause harm of any kind to individual participants – whether their physical, psychological, spiritual, emotional, social or financial well–being, or to their employability or professional relationships – or to their communities?

( ) Yes (•) No

Benefits/Risks

In answering the following questions (Q 37 – 45) please ensure that you address all issues relevant to the type of participants that will be involved in your research project. Refer for guidance to relevant chapters of the National Statement and other NHMRC guidelines as appropriate.

37. Does the research involve a practice or intervention which is an alternative to a standard practice or intervention?

(•) Yes ( ) No

Explain how the practice or intervention differs from standard practice or intervention:

The research involves treatment over the internet. While such offers of treatment are common, such treatment supported by a skilled clinician attending to the needs of the individual patient is very uncommon, even if desirable. There will be telephone contact with the participants. Diagnostic questionnaires will be administered over the phone by Dr Wims for panic patients, Ms Perini for depression patients, and Dr Mahoney for social phobia patients.

38. What expected benefits (if any) will this research have for other members of the population to which the participants belong?

If these Phase 2 trials are successful the Hospital would consider offering this type of treatment as a routine to the benefit of the wider community

39. What expected benefits (if any) will this research have for the wider community?

See above

40. What expected benefits (if any) will this research have for participants?

We would expect that the average participant would improve by 0.5–0.8 SDs on standardised measures of social phobia, depression and panic disorder.

41. Are there any risks to participants as a result of participation in this research project?

○ Yes ◉ No

42. Explain how the risks/burdens of participation are balanced by the benefits of the research.

Web based CBT is beneficial when people adhere to the program (Titov 2007). This research will foster adherence by good clinical guidance. We therefore expect people to benefit.

43. Is it possible that the research will involve the disclosure of unlawful conduct, or concealment of a crime, by individuals or definable groups?

○ Yes ◉ No

44. Explain how the dignity and wellbeing of participants takes precedence over the expected benefits to knowledge.

Participation is voluntary. Patients in the Phase 2a social phobia programs expressed themselves as very satisfied with the treatment and with their experience in treatment. We would expect that patients in this study would be no different.

45. Are there any other risks involved in this research? eg. to the research team, the organisation, others

○ Yes ◉ No

46. Is it anticipated that the research will lead to commercial benefit for the investigator(s) and or the research sponsor(s)?

○ Yes ◉ No

Monitoring

47. What mechanisms do the researchers intend to implement to monitor the conduct and progress of the research project?

Patient participation will be monitored on a daily basis. Monitoring will be facilitated by the course management software and will be the responsibility of the Principal Investigator.
Patients' progress is monitored by their feedback on homework activities and postings on forums. Patients who do not participate for more than two weeks will be contacted, first by email and then by phone. If they remain out of contact, and there is cause for concern, their general practitioner will be contacted.
Clinical outcomes of the participants are monitored by comparing pre– and post– questionnaire scores.

4. PROJECT SPECIFIC

Your responses to question 3 "Type of Research" and question 4 "Research participants" at Section 1 indicate that the HREC will require additional information which is specific to your research project. The following table indicates the question sets relating to the project that you will need to complete. If this is not correct please return to question 3 and 4 at Section 1 to amend your answer.

- 4.2. Clinical research (excluding under CTN/CTX scheme)

4.2. Clinical research (excluding under CTN/CTX scheme)

1. The study examines:

- [ ] The administration of a drug / medicine (includes a complementary / alternative medicine)
- [ ] The use of a medical device
- [x] Other

Describe briefly the type of study to be conducted:

An RCT of Clinician assisted computerised CBT vs computerised CBT vs waitlist

2. Provide the following details for the study protocol:

Protocol number:	N/A
Protocol version number:	2
Protocol version date:	02/04/2008 *(dd/mm/yyyy)*

3. Are there any protocol amendments appended to, or submitted with, the study protocol?

( ) Yes (•) No

4. Provide the following details for any amendments appended to, or submitted with, the clinical protocol:

5. Provide a statement addressing the following as may be applicable to the project.

a) Method of randomisation
b) Whether the hypothesis offers a realistic possibility that the intervention is at least as effective as standard treatment
c) Placebo use or non−treatment control group – justification, including alternative effective treatments and any risk of harm in the absence of treatment
d) Statistical justification, sample size calculations and method of analysis
e) Response variables and how treated
f) Endpoints
g) Details of contingencies and management of these.

a.) Using www.random.org

b.) Likely to be more effective than CCBT or wait list

c.) Placebo controls that do not deceive are difficult. This study compares the intervention with the usual computerised CBT and with wait list thus controlling for placebo response and natural history. NB after 13 weeks wait list subjects are offered active intervention.

d.) Sample size is powered to have an 80% chance of detecting differences at p<0.05 using a repeated mesures ANOVA.

e.) Response variables are the symptoms of the disorders and of stress and disability.

f.) Status at 13 weeks compared to the comparison groups, whether finished the six lesson course or not.

g.) Daily surveillance of patient progress, defined action: email, phone or GP contact if deterioration is suspected.

5. PARTICIPANTS

Participant description

1. How many participant groups are involved in this research project?

3

2. Expected total number of participants in this project at all sites?

405

3. Groups

Group 1

Group name for participants in this group: Panic disorder
Expected number of participants in this group: 105 (35 + 35 + 35)
Age range: 18+

Other relevant characteristics of this participant group:

All will meet criteria for panic disorder

Why are these characteristics relevant to the aims of the project?

This is a test of treatment for panic disorder

Group 2

Group name for participants in this group: Depression
Expected number of participants in this group: 195(65 + 65 + 65)
Age range: 18+

Other relevant characteristics of this participant group:

All will meet criteria for major depressive disorder

Why are these characteristics relevant to the aims of the project?

This is a test of treatment for depression

Group 3

Group name for participants in this group: Social phobia
Expected number of participants in this group: 105 (35 + 35 + 35)
Age range: 18+

Other relevant characteristics of this participant group:

All will meet criteria for social phobia

Why are these characteristics relevant to the aims of the project?

This is a test of treatment for social phobia

Your response to question 4 at Section 1 – "Research Participants" indicates that the following participant groups are excluded from your research. If this is not correct please return to question 4 at Section 1 to amend your answer.

- Children and/or young people (ie. <18 years)

- People with an intellectual or mental impairment

4. Explain why this group(s) of people is specifically excluded from the research project.

We need more data on the efficacy of these treatments in young people before inviting their participation. Future research will do this.

We need more data on the efficacy of these treatments in people with intellectual impairment before inviting their participation.

Participant experience

5. Provide a concise detailed description, in not more than 200 words, in terms which are easily understood by the lay reader, of what the participants will experience.

They will identify themselves as suffering from social phobia or panic disorder or depression and we will confirm this by questionnaire and by interview. The program will provide for the CaCCBT group, through illustrated story lines, homework exercises, postings on the forum and advice from the clinician, instruction about recovering from their disorder. They will then redo the questionnaires and receive supportive advice about their progress and additional needs for treatment, if any. The CCBT group will not receive the clinican support but will have access to lessons, the wait list group will receive nothing during that period. All three groups will do the exit questionnaires. Patients in the CCBT and waitlist groups will be offered CaCCBT at the end of the study if it proves to be useful.

Relationship of researchers to participants

6. Specify the nature of any relationship, existing or possible, between the research team or an organisation involved in the research and the potential participants.

The clinician will have, as a result of the patients application for treatment, a clinician–patient relationship. The patients will become patients of the Hospital.

7. Describe what steps, if any, will be taken to ensure that the relationship does not impair participants' free and voluntary consent and participation in the project.

Their consent remains free and voluntary and they can withdraw at any time.

8. Describe what steps, if any, will be taken to ensure that decisions about participation in the research do not impair any existing or foreseeable future relationship between participants and researcher or organisations.

This is distance treatment and few if any will have other needs for services from the Anxiety Disorders Clinic. They do however retain the right to apply for face to face treatment in the normal way.

Recruitment

9. What processes will be used to identify potential participants?

Publicity in the media. We have identified people with depression who volunteered for the shyness study. We expect an invited 702 "Drivetime" radio interview on 23rd April and consequent publicity to generate the people with social phobia, panic disorder and more people with depression needed for this study. We will encourage press about the project from the print media and from radio. We do not intend to place advertisements.

10. Describe how initial contact will be made with potential participants.

Interested people will log on to the web site www.climateclinic.tv, read about the study and decide to apply. Some 500 people responded to the limited publicity about the Phase 2 trial in social phobia. We still get some 5 or 10 per week.

11. Is it proposed to 'screen' or assess the suitability of the potential participants for the study?

(●) Yes ( ) No

How will this be done?

In terms of self identified main problem, confirmed by questionnaire and telephone interview.

12. Is an advertisement, e–mail, website, letter or telephone call proposed as the form of initial contact with potential participants?

(●) Yes ( ) No

Provide details and a copy of text/script:

The Shynessclinic.tv website is presently attracting about 7 enquiries per week and after the media interviews in April we anticipate 30 enquiries a week. There is no set text, we respond to the reporters or radio interviewers questions. The opening argument is that patients in the first two RCTs of Shynessclinic.tv made substantial improvements, equivalent to patients in face to face treatment in the SVH anxiety disorders clinic which has been benchmarked as one of the better clinics in the world.

13. If it became known that a person was recruited to, participated in, or was excluded from the research, would that knowledge expose the person to any disadvantage or risk?

( ) Yes (●) No

14. Will the research involve the intentional recruitment of any groups whose welfare, rights, beliefs, perceptions, customs or cultural heritage requires specific regard?

( ) Yes (●) No

Consent process

15. Will consent for participation in this research be sought from all participants?

(●) Yes ( ) No

16. Will there be participants who have capacity to give consent for themselves?

(●) Yes ( ) No

What mechanisms/assessments/tools are to be used, if any, to determine each of these participant's capacity to decide whether or not to participate?

The study will be described on the intake website. People will elect to continue or not. Only people whose responses to the questions are internally consistent will proceed to the second stage of diagnostic questionnaires and interview.

The diagnostic interview provides a further safeguard against wrongful inclusion.

17. Will there be participants who do not have capacity to give consent for themselves?

○ Yes ◉ No

The following questions relate to participants who are able to provide consent and also to participants for whom consent may be provided by a person with legal authority to do so.

When answering these questions you need to describe any differences in the processes followed, or the documentation used, for different groups of participants in your proposal, e.g. processes and documentation for users of facilities/services will differ from those for providers of those facilities/services. Where your proposal involves participants with an intellectual or mental impairment, or people in dependent relationships, additional questions about their consent appear at Section 6 (Q8−9 and Q11−14 respectively).

18. Describe the consent process, ie how participants or those deciding for them will be informed about, and choose whether or not to participate in, the project.

The study will be described on the intake website. People will elect to continue or not, screening as to suitability will be automated. Only people whose responses to the questions match criteria and are internally consistent with the diagnostic criteria will proceed to the second stage of diagnostic questionnaires and interview. Participants registering for final consideration will read the information sheet and sign a consent form and mail that to the investigators. This will trigger the phone interview in which the diagnosis will be confirmed, questions about the study answered, and an offer of treatment made.

19. If a participant or person on behalf of a participant chooses not to participate, are there specific consequences of which they should be made aware, prior to making this decision?

No

20. If a participant or person on behalf of a participant chooses to withdraw from the research, are there specific consequences of which they should be made aware, prior to giving consent?

No

21. Specify the nature and value of any proposed incentive/payment (eg. movie tickets, food vouchers) or reimbursement (eg travel expenses) to participants.

None

22. Explain why this offer will not impair the voluntary nature of the consent, whether by participants' or persons deciding for their behalf.

NA

23. Provide the name and/or position of the contact person for any concerns in relation to the ethical conduct of the research / complaints process?

Sarah Charlton. Executive Officer, St Vincent's Human Research Ethics Committee.

24. Will a participant or person on their behalf who withdraws from the research be able to withdraw data about the participant?

(•) Yes ( ) No

6. Participants Specific

Research conducted in Australia involving persons whose primary language is other than English (LOTE)

You have indicated that the project involves persons whose primary language is other than English (LOTE)

1. Describe what steps will be taken to ensure each participant's free and voluntary consent and participation in the project given that the person's language is other than English?

That their primary language is not English is not of concern. They will however need to be able to read and write English at a School Certificate level.

2. In what language(s) will the research be conducted?

- [x] English
- [ ] Other

3. Will participants be provided with written information in the language in which the research will be conducted?

(●) Yes ( ) No

7. RESOURCES

Project Funding / Support

1. Indicate how the project will be funded? Indicate whether funding is confirmed or sought and whether there will be a budget shortfall.

Funding	Confirmed or Sought?	
External competitive grant	◯ Confirmed ◯ Sought ◯ Not Sought	*% of project:*
Internal competitive grant	◯ Confirmed ◯ Sought ◯ Not Sought	*% of project:*
Sponsor	◯ Confirmed ◯ Sought ◯ Not Sought	*% of project:*
By researcher's department / organisation	◉ Confirmed ◯ Sought ◯ Not Sought	*% of project:* 70
Other	◯ Confirmed ◉ Sought ◯ Not Sought	*% of project:* 30
Shortfall	◯ Confirmed ◯ Sought ◯ Not Sought	*% of project:*

1a. External competitive grant

Name of granting body / name of grant:

Research title on grant:

Grant number assigned by granting body (if known):

Indicate the extent to which the scope of the grant and the scope of this HREC application are aligned:

Comments:

1b. Internal competitive grant

Name of granting body / name of grant:

Research title on grant:

Grant number assigned by granting body (if known):

Indicate the extent to which the scope of the grant and the scope of this HREC application are aligned:

Comments:

1c. Sponsor

Name of sponsor(s):

Describe the nature of the relationship between the sponsor(s) and the research project:

1d. By researcher's department / organisation

Name of department(s)/organisation:	St Vincent's Mental Health Service

1e. Other

Name of funding provider(s):	St Vincent's is establishing a research foundation. We have applied for a research assistants salary to manage the project. We will forward details on any appointee when that occurs.
Describe the nature of the relationship between the funding provider and the research project:	No relationship

1f. Shortfall

What are the implications of the funding shortfall and how might this affect participants?	CRUfAD has trust funds that could cover any shortfall

2. Will the project be supported in other ways eg. in-kind support/equipment by an external party eg. sponsor?

○ Yes ◉ No

Duality of Interest

3. Describe any commercialisation or intellectual property implications of the funding/support arrangement.

The CaCCBT and the CCBT programs are the property of St Vincent's Hospital

4. Does the funding/support provider(s) have a financial interest in the outcome of the research?

◉ Yes ○ No

Describe the interest:

The hospital owns it and employs the staff

Do you consider the funding/support arrangement constitutes:

- ☐ a potential conflict of interest
- ☐ a potential duality of interest
- ☑ no ethical issue

Provide an explanation:

The hospital treats people, this is a program to do that

5. Does any member of the research team have any affiliation with the provider(s) of funding/support, or a financial interest in the outcome of the research?

(•) Yes ( ) No

Describe affiliation(s) and/or interest(s):

All five researchers are clinicians affiliated with the Hospital

Do you consider the relationship between the research team and the funding/support provider constitutes:

- [ ] a potential conflict of interest
- [ ] a potential duality of interest
- [x] no ethical issue

Provide an explanation:

It is their job

6. Does any other individual or organisation have an interest in the outcome of this research?

( ) Yes (•) No

8. APPROVALS

Ethical review

Some HRECs may require researchers to provide information additional to that contained in a NEAF proposal. For this reason, it is prudent to check whether the HRECs to whom you propose to submit this proposal require additional information.

1. To how many Australian HRECs (representing site organisations or the researcher's organisation) is it intended that this research proposal be submitted?

1

A list of NHMRC registered Human Research Ethics Committees (HRECs), along with their institutional affiliations and contact details is available on the NHMRC website at the following web address: http://www.nhmrc.gov.au/ethics/human/hrecs/information.htm#a4.

2. HRECs

HREC 1

Name of HREC:

St Vincent's Hospital Human Research Ethics Committee (EC00140)

Provide the start and finish dates for the research for which this HREC is providing ethical review:

Anticipated start date or date range: 10/04/2008 *(dd/mm/yyyy)*
Anticipated finish date or date range: 31/12/2009 *(dd/mm/yyyy)*

For how many sites at which the research is to be conducted will this HREC provide ethical review?

1

3. Have you previously submitted an application, whether in NEAF of otherwise, for ethical review of this research project to any other HRECs?

○ Yes ◉ No

4. HRECs

Declarations And Signatures

Applicant / Principal Researchers (including students where permitted)

Project Title (in full):	Distance treatment III: Randomised controlled trial in social phobia, panic and depression
HREC to which this application is made:	St Vincent's Hospital Human Research Ethics Committee (EC00140)
HREC Reference number:	08/SVH/36

I/we certify that:

- All information is truthful and as complete as possible.
- I/we have had access to and read the National Statement on Ethical Conduct in Research InvolvingHumans.
- The research will be conducted in accordance with the National Statement.
- The research will be conducted in accordance with the ethical and research arrangements of the organisations involved.
- The research will be conducted in accordance with the ethical and research arrangements of the organisations involved.
- I/we have consulted any relevant legislation and regulations, and the research will be conducted in accordance with these.
- I/we will immediately report to the HREC anything which might warrant review of the ethical approval of the proposal (NS 2.37), including:
- serious or unexpected adverse effects on participants;
- proposed changes in the protocol; and
- unforseen events that might affect continued ethical acceptability of the project.
- I/we will inform the HREC, giving reasons, if the research project is discontinued before the expected date of completion (NS 2.38);
- I/we will not continue the research if ethical approval is withdrawn and will comply with any special conditions required by the HREC (NS. 2.45);
- I/we will adhere to the conditions of approval stipulated by the HREC and will cooperate with HREC monitoring requirements. At a minimum annual progress reports and a final report will be provided to the HREC.

Applicant / Principal Researchers

Professor John Gavin Andrews<br>UNSW at St Vincent's Hospital, Sydney	....<br>Signature	...../ ...../ ....<br>Date
Professor John Gavin Andrews<br>UNSW at St Vincent's Hospital, Sydney	....<br>Signature	...../ ...../ ....<br>Date
Dr Nickolai Titov<br>UNSW at St Vincent's Hospital, Sydney	....<br>Signature	...../ ...../ ....<br>Date

Associate Researchers

Ms Sarah Perini<br>St Vincent's Hospital, Sydney	....<br>Signature	...../ ...../ ....<br>Date

Dr Edward Wims<br>St Vincent's Hospital	....<br>Signature	...../ ...../ ....<br>Date
Dr Alison Mahoney<br>St Vincent's Hospital	....<br>Signature	...../ ...../ ....<br>Date

Supervisor(s) of student(s)

Project Title (in full):	Distance treatment III: Randomised controlled trial in social phobia, panic and depression
HREC to which this application is made:	St Vincent's Hospital Human Research Ethics Committee (EC00140)
HREC Reference number:	08/SVH/36

I/we certify that:

- I/we will provide appropriate supervision to the student to ensure that the project is undertaken in accordance with the undertakings above;
- I/we will ensure that training is provided necessary to enable the project to be undertaken skilfully and ethically.

.... Signature	...../ ...../ .... Date
.... Signature	...../ ...../ .... Date
.... Signature	...../ ...../ .... Date
.... Signature	...../ ...../ .... Date
.... Signature	...../ ...../ .... Date

Heads of departments/schools/research organisation

Project Title (in full):	Distance treatment III: Randomised controlled trial in social phobia, panic and depression
HREC to which this application is made:	St Vincent's Hospital Human Research Ethics Committee (EC00140)
HREC Reference number:	08/SVH/36

I/we certify that:

- I/we are familiar with this project and endorse its undertaking;
- the resources required to undertake this project are available;
- the researchers have the skill and expertise to undertake this project appropriately or will undergo appropriate training as specified in this application.

....
Title First Name Surname

....
Position Organisation Name

..../ ...../ ....
Signature Date

10. Attachments

List of Attachments

Core Attachments	Attachments which may be required/appropriate
Recruitment/invitation	Copy of advertisement, letter of invitation etc
Participant Information	Copy or script for participant<br>Copy or script for parent, legal guardian or person responsible as appropriate
Consent Form	Copy for participant<br>For parent, legal guardian or person responsible as appropriate<br>For, optional components of the project eg. genetic sub study
Peer review	Copy of peer review report or grant submission outcome
HREC approvals	Copy of outcome of other HREC reviews

Attachments specific to project or participant group	Attachments which may be required/appropriate
People whose primary language is other than English (LOTE)	English translation of participant information/consent forms
Survey instrument / questionnaire / diary	Copy of instrument/questionnaire/diary pro forma
Internet / web based research	Copy of web content / questionnaire
Interviews – telephone	Copy of script/outline

Participant information elements

Core Elements
Provision of information to participants about the following topics should be considered for all research projects.

**Core Elements**	**Issues to consider in participant information**
About the project	Full title and / or short title of the project<br>Plain language description of the project<br>Purpose / aim of the project and research methods as appropriate<br>Demands, risks, inconveniences, discomforts of participation in the project<br>Outcomes and benefits of the project<br>Project start, finish, duration
About the investigators / organisation	Researchers conducting the project (including whether student researchers are involved)<br>Organisations which are involved / responsible<br>Organistions which have given approvals<br>Relationship between researchers and particpants and organisations
Participant description	How and why participants are chosen<br>How participants are recruited<br>How many participants are to be recruited
Participant experience	What will happen to the particant, what will they have to do, what will they experience?<br>Benefits to individual, community, and contribution to knowledge<br>Risks to individual, community<br>Consequences of participation
Participant options	Alternatives to participation<br>Whether participation may be for part of project or only for whole of project<br>Whether any of the following will be provided: counselling, post research follow−up, or post research access to services, equipment or goods
Participants rights and responsibilities	That participation is voluntary<br>That participants can withdraw, how to withdraw and what consequences may follow<br>Expectations on participants, consequences of non−compliance with the protocol<br>How to seek more information<br>How to raise a concern or make a complaint
Handling of information	How information will be accessed, collected, used, stored, and to whom data will be disclosed<br>Can participants withdraw their information, how, when<br>Confidentiality of information<br>Ownership of information<br>Subsequent use of information<br>Storage and disposal of information
Unlawful conduct	Whether researcher has any obligations to report unlawful conduct of participant
Financial issues	How the project is funded<br>Declaration of any duality of interests<br>Conspensation entitlements<br>Costs to participants<br>Payments, reimbursements to participants

	Commercial application of results
Results	What will particpants be told, when and by whom<br>Will individual results be provided<br>What are the consequences of being told or not being told the results of research<br>How will results be reported / published<br>Ownership of intellectual property and commercial benefits
Cessation	Circumstances under which the participation of an individual might cease<br>Circumstances under which the project might be terminated

Research Specific Elements
Provision of information to participants about the following topics should be considered as may be relevant to the research project.

Specific to project or participant group	Additional issues to consider in participant information

Site–Specific Assessment (SSA) Form

- *This form must be completed by the Principal Investigator responsible for the research project at this site.*
- *The completed form must be forwarded to the site's Research Governance Officer for authorisation and the signature of the Chief Executive/or delegate.*

SSA is a component of research governance and involves assessment of the suitability of the site and the Investigator(s) for the proposed research.

The data in this box is populated from NEAF:

Short title and version number:
Distance treatment III: RCT in social phobia, panic and depression

Name/ID of HREC reviewing the research project:
St Vincent's Hospital Human Research Ethics Committee (EC00140)

HREC Application Reference Number: 08/SVH/36

Name of organisation responsible for SSA:

SSA reference

1. Project Title (in full): *(populated from NEAF)*

Distance treatment III: Randomised controlled trial in social phobia, panic and depression

Project summary: *(populated from NEAF)*

Provide a brief description (half page) of the project details to enable the research governance officer to understand the nature and impact of the research project at the research site.

Many people with social phobia, panic disorder or depressive disorder do not access treatment. Internet based therapies exist but adherence is poor without clinician guidance. We have modified existing patient education internet programs used by GPs as the basis for clinician guided computerised cognitive behaviour therapy programs (the interventions) for adults with social phobia, panic disorder or with depression.

This phase 2b study will recruit 105 people with panic disorder, 195 people with depression, and 105 people with social phobia and randomly allocate them to clinician assisted computerised cognitive behaviour therapy (CaCCBt) or computerised cognitive behaviour therapy (CCBT) or to remaining on a waitlist with the prospect of active treatment after 13 weeks. We will measure changes in symptom level from the beginning of the the 6–10 week course of treatment and one week after the conclusion of treatment. Measurement in the control group will be matched to this. We will also measure adherence to the lessons, homework and forum; satisfaction with the clinician's input and satisfaction with the mode of treatment generally.

2. Give the name of the project site to which this SSA applies.

St Vincent's Hospital

3. Research Personnel (at your site only)
Provide details of researchers' qualifications, expertise/skills and experience in areas related to the research project.

Principal Investigator

Title:	Forename/Initials:	Surname:
Professor	John Gavin	Andrews

Mailing Address:	299 Forbes St
Suburb/Town:	Darlinghurst
State:	NSW
Postcode:	2010
Country:	Australia
Organisation:	UNSW at St Vincent's Hospital
Department*:	
Position:	Director
E−mail:	gavina@unsw.edu.au
Phone (BH):	8382 1726
Phone (AH)*:	
Mobile*:	
Pager*:	
Fax:	8382 1721

Describe the qualifications, expertise and experience of the principal researcher relevant to this project.

Qualifications:	MD
Expertise:	Written books and papers on anxiety and depression
Experience:	Directed anxiety disorders clinic
Role in research project:	Director

Is evidence of current Professional Medical Registration attached? ○ Yes ○ No ◉ N/A
(Not applicable in NSW and Queensland)

Is a *Curriculum Vitae* attached (2 page maximum). ◉ Yes ○ No ○ N/A

Associate Investigators

Investigator 1

Title:	Forename/Initials:	Surname:
Dr	Nickolai	Titov

Mailing Address:	299 Forbes St
Suburb/Town:	Darlinghurst
State:	NSW
Postcode:	2010
Country:	Australia
Organisation:	UNSW at St Vincent's Hospital

Department*:
Position: Senior Lecturer
E–mail: nickt@unsw.edu.au
Phone (BH): 8382 1732
Phone (AH)*:
Mobile*:
Pager*:
Fax: 8382 1721

Describe the qualifications, expertise and experience of the associate researcher relevant to this project.

Qualifications: PhD
Expertise: Clinical Psychologist with special interest in internet therapy
Experience: Developed CaCBT model
Role in research project: Advice to Clinicians

Is evidence of current Professional Medical Registration attached? ( ) Yes ( ) No (•) N/A
(Not applicable in NSW and Queensland)

Is a *Curriculum Vitae* attached (2 page maximum). (•) Yes ( ) No ( ) N/A

Investigator 2

Title:	Forename/Initials:	Surname:
Ms	Sarah	Perini

Mailing Address: 299 Forbes St

Suburb/Town: Darlinghurst
State: NSW
Postcode: 2010
Country: Australia
Organisation: St Vincent's Hospital, Sydney
Department*:
Position: Clinical psychologist
E–mail: sperini@stvincents.com.au
Phone (BH):
Phone (AH)*:
Mobile*:
Pager*:
Fax:

Describe the qualifications, expertise and experience of the associate researcher relevant to this project.

Qualifications: M Clinical Psychol
Expertise: Experienced clinician with expertise in cognitive behaviour therapy for depression
Experience: Designed the CaCCBT program
Role in research project: Clinician for Depression group

Is evidence of current Professional Medical Registration attached? ( ) Yes ( ) No (•) N/A
(Not applicable in NSW and Queensland)

Is a *Curriculum Vitae* attached (2 page maximum). (•) Yes ( ) No ( ) N/A

Investigator 3

	Title:	Forename/Initials:	Surname:
	Dr	Edward	Wims

Mailing Address: 299 Forbes St

Suburb/Town: Darlinghurst
State: NSW
Postcode: 2010
Country: Australia
Organisation: St Vincent's Hospital
Department*:
Position: Senior registrar
E-mail: ewims@stvincents.com.au
Phone (BH):
Phone (AH)*:
Mobile*:
Pager*:
Fax:

Describe the qualifications, expertise and experience of the associate researcher relevant to this project.

Qualifications:	MB Bch BAO, MRCPsych
Expertise:	Trained in CBT for panic disorder
Experience:	Developed the Panic CaCCBT program
Role in research project:	Clinician for panic patients

Is evidence of current Professional Medical Registration attached? ○ Yes ○ No ◉ N/A
(Not applicable in NSW and Queensland)

Is a *Curriculum Vitae* attached (2 page maximum). ◉ Yes ○ No ○ N/A

Investigator 4

	Title:	Forename/Initials:	Surname:
	Dr	Alison	Mahoney

Mailing Address: 299 Forbes ST

Suburb/Town: Darlinghurst
State: NSW
Postcode: 2010
Country: Australia
Organisation: St Vincent's Hospital
Department*:
Position: Clinical Psychologist
E-mail: amahoney@stvincents.com.au
Phone (BH): 83821720
Phone (AH)*:
Mobile*:
Pager*:
Fax: 83821721

Describe the qualifications, expertise and experience of the associate researcher relevant to this project.

Qualifications: PsyD
Expertise: Clinical Psychologist with experience in cognitive behaviour therapy for anxiety disorders
Experience: Two years experience treating people with social phobia
Role in research project: Clinician for social phobia group
Is evidence of current Professional Medical Registration attached? *(Not applicable in NSW and Queensland)* ( ) Yes ( ) No (•) N/A
Is a *Curriculum Vitae* attached (2 page maximum). (•) Yes ( ) No ( ) N/A

Contact person for this research project

Title:	Forename/Initials:	Surname:
Professor	John Gavin	Andrews

Mailing Address: 299 Forbes St

Suburb/Town: Darlinghurst
State: NSW
Postcode: 2010
Country: Australia
Organisation: UNSW at St Vincent's Hospital
Department*:
Position: Director
E-mail: gavina@unsw.edu.au
Phone (BH): 8382 1726
Phone (AH)*:
Mobile*:
Pager*:
Fax: 8382 1721

4. Training
Will any of the researchers require extra training to enable their participation in this project?

( ) Yes (•) No

5. Recruitment of Participants
What is the proposed number of participants to be recruited at this site?

405

6. Participant details
What categories of people will be recruited? *(e.g. children and young people, people with an intellectual or mental impairment, people highly dependent on medical care, people in dependent or unequal relationships, Aboriginal & Torres Strait Islander people, persons in custody, etc)*

Adults with self identified social phobia, panic disorder or depression

7. What additional time and resources above their routine duties will be required of the research team throughout the research project?

	Name	Department/location	Additional time spent (hours/week)
1	Edward Wims	Anxiety Disorders	0
2	Sarah Perini	Anxiety Disorders	0
3	Alison Mahoney	Anxiety Disorders	0
4		This internet therapy is part of their job description. Evaluation should be part of all good clinical care.	

8. Anticipated start and finish dates for the research project?

Start date: 10/04/2008 *(dd/mm/yyyy)*
Finish date: 31/12/2009 *(dd/mm/yyyy)*
Duration (Months): 21

9. Departments and services involved in research

List and specify the departments/locations involved in the research, which are part of this site.

	Department/location	Name of responsible person
1	Anxiety Disorders	JG Andrews

A signed declaration from the Head of Department / organisation must be attached (see #14. Declarations).

10. Study budget

An explanation of how the research project will be funded at the site must be provided to ensure adequate financial arrangements are planned. To assess the financial impact of the research any costs incurred by the organisation should be provided.

Type of funding	Source of funding	Amount ($/year or $/participant)
Commercially Sponsored		
Sponsored, other *(e.g. collaborative groups)*		
External funding *(e.g.NHMRC, Foundations, etc)*		
Internal/Departmental funding		

Give details of the type and name of the funding organisation

Other financial, material and capital support.

Infrastructure charge	
Supply of drug(s)	
Loan of equipment	
Other	

Give details of support given

Which organisation will receive and manage this funding and/or will be the Administering Organisation?

Organisation: NA

Details of contact person

Title: Forename/Initials: Surname:

Position:

Department:

Mailing Address:

Suburb/Town:

State:

Postcode:

Country:

E−mail:

Phone (BH):

Phone (AH)*:

Mobile*:

Pager*:

Fax:

Insert the account number(s)/cost centre details into which funds are to be deposited:

Give full address for correspondence

11. Site−specific policies

For organisations that have site specific policies. *(e.g. Wording related to the use of contraception in participant information and consent documents.)*

This must not be used by the site to require re−review of the consent documents by the local HREC

Does the research comply with site−specific policies/requirements?

(●) Yes ( ) No

12. Clinical trials information
If the study is a clinical trial the following sections must be completed.

12a. Is the research project being conducted under the Clinical Trial Notification (CTN) or Clinical Trial Exemption (CTX) schemes?

○ Yes ◉ No

See Standard Operating Procedures.

12b. Is the Medicines Australia Standard Indemnity Form(s), signed by the sponsor attached?

○ Yes ○ No ◉ N/A

If no or N/A please give an explanation:

Not a medicine

12c. Is evidence of adequate insurance cover attached?

○ Yes ◉ No ○ N/A

If no or N/A please give an explanation:

Treasury Managed Fund will cover as they do all treatment in public hospitals

12d. Is the Medicines Australia Standard Clinical Trial Agreement(s), signed by the sponsor attached?

○ Yes ○ No ◉ N/A

If no or N/A please give an explanation:

Not a medicine

13. Biosafety, chemical and radiation safety

It may be necessary for research organisations to complete notification, registration or licence requirements for research involving biosafety, regulatory issues and/or radiation. If so, evidence of this is required.

If "yes" is ticked below, appropriate documentation of approval must be attached or forwarded to the site's Research Governance Officer when available.

1. Is Institutional Biosafety Committee (IBC) notification and/or licence application to the Office of the Gene Technology Regulator (OGTR) for approval of genetically modified organisms required? ○ Yes ○ Attached ◉ No

2.. Is committee approval of chemical safety required (drugs/pharmacy committee)? ○ Yes ○ Attached ◉ No

3.. Will the project require NHMRC Gene and Related Therapies Research Advisory Panel (GTRAP) assessment? ○ Yes ◉ No

4.. Will the project require application for a licence to the NHMRC Licensing Committee to conduct embryo research? ○ Yes ◉ No

5.. For projects where Australian Radiation Protection and Nuclear Safety Agency (ARPANSA) Code compliance is required, is additional State-specific radiation safety approval and registration required? ○ Yes ◉ No

See Standard Operating Procedures for additional details.

Declarations

(a) Declaration by the Principal Investigator and Associate Investigator(s)

HREC Reference number:	08/SVH/36
Project Title (in full):	Distance treatment III: Randomised controlled trial in social phobia, panic and depression
Principal Investigator:	Professor John Gavin Andrews

1. I declare the information in this form is truthful and accurate to the best of my knowledge and belief and I take full responsibility at this site.
2. I will only start this research project after obtaining authorisation from the site and approval from the responsible Human Research Ethics Committee (HREC);
3. I accept responsibility for the conduct of this research project according to the principles of the NHMRC *National Statement on Ethical Conduct in Research*.
4. I undertake to conduct this research project in accordance with the protocols and procedures as approved by the HREC and the ethical and research arrangements of the organisation(s) involved.
5. I undertake to conduct this research in accordance with relevant legislation and regulations.
6. I agree to comply with the requirements of adverse or unexpected event reporting as stipulated by the HREC and NHMRC
7. I will adhere to the conditions of approval stipulated by the HREC and will cooperate with HREC monitoring requirements.
8. I will inform the HREC and the research governance officer if the research project ceases before the expected date. I will discontinue the research if the HREC withdraws ethical approval.
9. I will adhere to the conditions of authorisation stipulated by the authorising authority at the site where I am Principal Investigator. I will discontinue the research if the authorising authority withdraws authorisation at the site where I am Principal Investigator.
10. I understand and agree that study files and documents and research records and data may be subject to inspection by the HREC, research governance officer, the sponsor or an independent body for audit and monitoring purposes.
11. I understand that information relating to this research, and about me as a researcher, will be held by the HREC, research governance officer, and on the Research Ethics Database (RED). This information will be used for reporting purposes and managed according to the principles established in the Privacy Act 1988 (Cth) and relevant laws in the States and Territories of Australia.

Signature of Principal Investigator: ....

Print Name: Professor John Gavin Andrews Date: 13/02/2008

Signature of Associate Investigator: ....

Print Name: Dr Nickolai Titov Date: 13/02/2008

Signature of Associate Investigator: ....

Print Name: Ms Sarah Perini Date: 13/02/2008

Signature of Associate Investigator: ....

Print Name: Dr Edward Wims Date: 13/02/2008

Signature of Associate Investigator: ....

Print Name: Dr Allison Mahoney Date: 13/02/2008

(b) Declaration by Head of Department *(or Divisional Director or other authority) where the Principal Investigator will do the research.

HREC Reference number:	08/SVH/36
Project Title (in full):	Distance treatment III: Randomised controlled trial in social phobia, panic and depression
Principal Investigator:	Professor John Gavin Andrews

- I certify that I have read the research project application named above.
- I certify that I have discussed this research project and the resource implications for this Department, with the Principal Investigator.
- I certify that all researchers/students from my Department involved in the research project have the skills, training and experience necessary to undertake their role.
- I certify that there are suitable and adequate facilities and resources for the research project to be conducted at this site.
- My signature indicates that I support this research project being carried out using such resources.

Name of Head of Department
(or appropriate person):

Name of Department
(or relevant section):

Signature: ....

Print Name: Date:

** Where an investigator is also Head of Department, certification must be sought from the person to whom the Head of Department is responsible. Investigators must not approve their own research on behalf of their Department.*

(c) Declaration by Head of Supporting Department

This form is to be completed by the Head of any Department that is providing support or services to the research project, but which does not have any member(s) on the research team.

HREC Reference number:	08/SVH/36
Project Title (in full):	Distance treatment III: Randomised controlled trial in social phobia, panic and depression
Principal Investigator:	Professor John Gavin Andrews

I have discussed this project with the Principal Investigator and have read the research project. I am *(tick whichever applies)*

◯ able to perform the investigations/services indicated, within the present resources of the Department;

◯ able to perform the investigations/services indicated, if the following financial assistance is provided:

◯ unable to undertake the investigations/services indicated, on the following grounds:

Name: Date:

Signature: ....

Department:

(d) Declaration by the Authority for Data Provision

This form is to be completed by the person authorised to provide data services for research projects.

HREC Reference number:	08/SVH/36
Project Title (in full):	Distance treatment III: Randomised controlled trial in social phobia, panic and depression
Principal Investigator:	Professor John Gavin Andrews

I have considered this proposal and consulted the appropriate personnel and I confirm that I have seen all relevant documents that are required. The Department(s) is *(tick whichever applies)*

○ able to confirm that the data services indicated will be provided, within the present resources;

○ able to confirm that the data services indicated will be provided, if the following financial assistance is provided:

○ unable to provide data services indicated, on the following grounds:

I certify that I will give due regard to any ethical conditions imposed by the approving HREC when deciding whether, and in what form, I will release data to the investigator.

Name: Date:

Position:

Signature: ....

Department:

(e) Recommendation by the Research Governance Office

HREC Reference number:	08/SVH/36
Project Title (in full):	Distance treatment III: Randomised controlled trial in social phobia, panic and depression
Principal Investigator:	Professor John Gavin Andrews

The Site−Specific Assessment (SSA) form for the above research project has been completed (with all attachments).

SSA authorisation is:

- ○ Recommended
- ○ Not recommended
- ○ Requires Chief Executive/delegate consideration

If not recommended or requires Chief Executive/delegate consideration, give reasons.

Research Governance Officer Name (or equivalent):

Date:

Signature: ....

(f) Authorisation by Chief Executive (or delegate)

HREC Reference number:	08/SVH/36
Project Title (in full):	Distance treatment III: Randomised controlled trial in social phobia, panic and depression
Principal Investigator:	Professor John Gavin Andrews

This research is: ◯ authorised ◯ not authorised

Specify, conditions applying to authorisation or reasons for not authorising.

My signature indicates that I authorise/ do not authorise this research project to commence at this site.

Name of Chief Executive (or delegate):
Name of Organisation:

Date:

Signature: ....

Checklist

Please complete the checklist with Yes: No: NA (Not Applicable). Include this checklist with the SSA Form.

HREC Reference number:	08/SVH/36
Project Title (in full):	Distance treatment III: Randomised controlled trial in social phobia, panic and depression
Principal Investigator:	Professor John Gavin Andrews

	Person Completing Form	Office Use Only
Has a CV been attached for each researcher?	(•) Yes ( ) No ( ) N/A	( ) Yes ( ) No ( ) N/A
Have you attached proof of Professional Medical Registration? (NA in NSW and Queensland)	( ) Yes ( ) No (•) N/A	( ) Yes ( ) No ( ) N/A
Has a contact person for this research project been nominated?	(•) Yes ( ) No ( ) N/A	( ) Yes ( ) No ( ) N/A
Have you completed all financial details in #10?	( ) Yes ( ) No (•) N/A	( ) Yes ( ) No ( ) N/A
Has a copy of the HREC approval letter been provided?	( ) Yes ( ) No (•) N/A	( ) Yes ( ) No ( ) N/A
Has a copy of the ethics application form been provided?	(•) Yes ( ) No ( ) N/A	( ) Yes ( ) No ( ) N/A
Has a copy of the protocol been provided?	(•) Yes ( ) No ( ) N/A	( ) Yes ( ) No ( ) N/A
Has a copy of the Investigator's Brochure/drug information/device information been provided?	( ) Yes ( ) No (•) N/A	( ) Yes ( ) No ( ) N/A
Are all Participant Information and Consent Form(s) attached and show the name of the Institution and contact details of the Principal Site Investigator? <u>The version number, standard organisation name and date should be in the footer.</u>	(•) Yes ( ) No ( ) N/A	( ) Yes ( ) No ( ) N/A
Has a copy of advertising been provided?	( ) Yes ( ) No (•) N/A	( ) Yes ( ) No ( ) N/A
Has a copy of any questionnaires been provided?	(•) Yes ( ) No ( ) N/A	( ) Yes ( ) No ( ) N/A
Has a copy of any other document, which will be given to research participants been provided? Eg: identification card, patient diary	( ) Yes ( ) No (•) N/A	( ) Yes ( ) No ( ) N/A
If a clinical trial, are CTN/CTX forms, **signed** by the approving HREC and Principal Site Investigator, attached?	( ) Yes ( ) No (•) N/A	( ) Yes ( ) No ( ) N/A
Is the Medicines Australia Standard Indemnity Form, **signed** by the sponsor, attached?	( ) Yes ( ) No (•) N/A	( ) Yes ( ) No ( ) N/A
Is evidence of adequate insurance cover attached?	( ) Yes ( ) No (•) N/A	( ) Yes ( ) No ( ) N/A
Is the Medicines Australia Standard Clinical Trial Agreement(s), **signed** by the sponsor, attached?	( ) Yes ( ) No (•) N/A	( ) Yes ( ) No ( ) N/A

Has evidence of Biosafety approval been provided?	○ Yes ○ No (•) N/A	○ Yes ○ No ○ N/A
Has committee approval of chemical safety been provided (pharmacy/drug)?	○ Yes ○ No (•) N/A	○ Yes ○ No ○ N/A
Has evidence of an application for NHMRC Gene Related Therapies assessment been provided?	○ Yes ○ No (•) N/A	○ Yes ○ No ○ N/A
Has evidence of an application for a licence to the NHMRC Licensing Committee, to conduct embryo research, been provided?	○ Yes ○ No (•) N/A	○ Yes ○ No ○ N/A
Has evidence of Radiation Safety approval been provided?	○ Yes ○ No (•) N/A	○ Yes ○ No ○ N/A
Have you included any other site−specific policy documents required by the Institution(s) at which you intend to conduct your research?	(•) Yes ○ No ○ N/A	○ Yes ○ No ○ N/A
Is a "Declaration by Principal Investigator" signed and attached?	(•) Yes ○ No ○ N/A	○ Yes ○ No ○ N/A
Is a "Declaration by Head of Department" signed and attached?	(•) Yes ○ No ○ N/A	○ Yes ○ No ○ N/A
Is a "Declaration by Head of Supporting Department" signed and attached for each supporting Department (if applicable)?	○ Yes ○ No (•) N/A	○ Yes ○ No ○ N/A
Is a "Declaration by the Authority for Data Provision" signed and attached (if applicable)?	○ Yes ○ No (•) N/A	○ Yes ○ No ○ N/A
Are all pages (including attachments) numbered and dated in the footer?	(•) Yes ○ No ○ N/A	○ Yes ○ No ○ N/A
